# *Cysticercus bovis* in slaughtered cattle in upper Egypt: implications for food safety

**DOI:** 10.1186/s12917-025-04768-y

**Published:** 2025-05-15

**Authors:** Nady Khairy Elbarbary, Ahmed Gareh, Maha Abdelhaseib, Ahmed Fotouh, Neveen M. Abdelmotilib, Mohammed Fathy Ragab, Mohamed K. Dandrawy

**Affiliations:** 1 Food Hygiene and Control Department, Faculty of Veterinary Medicine, Aswan University, Aswan, 81528 Egypt; 2 Parasitology Department, Faculty of Veterinary Medicine, Aswan University, Aswan, 81528 Egypt; 3 Food Hygiene, Safety and Technology Department, Faculty of Veterinary Medicine, Assiut University, Assiut, 71526 Egypt; 4 Pathology and Clinical Pathology Department, Faculty of Veterinary Medicine, New Valley University, El-Kharga, Egypt; 5 Food Technology Department, Arid Lands Cultivation Research Institute (ALCRI), City of Scientific Research and Technological Applications (SRTA-CITY), New Borg El-Arab City, 21934 Egypt; 6 Medical Parasitology Department, Faculty of Medicine, Damietta University, Damietta, 34517 Egypt; 7 Food Hygiene and Control Department, Faculty of Veterinary Medicine, South Valley University, Qena, 83522 Egypt

**Keywords:** *Cysticercus bovis HDP2 gene*, Implications of food safety, Postmortem inspection, Risk factors, *Taenia saginata*

## Abstract

**Background:**

Bovine cysticercosis is regarded as an essential food safety concern, causing human taeniasis, as well as a significant economic worry, as infected carcasses are condemned, frozen, and downgraded. It is caused by *Cysticercus bovis* (*C. bovis*), which is the larval stage of *Taenia saginata* that inhabits the small intestine of man. In the two-host life cycle, humans are the definitive hosts, and cattle are the intermediate hosts. Therefore, the current research aims to study the prevalence of *C. bovis* in slaughtered cattle in Aswan province, Upper Egypt, by using both macroscopic and molecular techniques.

**Methods:**

A cross-sectional study on *C. Bovis* in slaughtered cattle was conducted from July 2023 to April 2024 at several central slaughterhouses (Edfu, Kom Ombo, Daraw, Aswan, and Abu Simbel) in Aswan province, Egypt, to ascertain the prevalence of *C. bovis* in 47,763 slaughtered cattle through routine daily antemortem and postmortem inspections; histopathological inspection and molecular analysis were implemented to verify the findings.

**Results:**

Out of 47,763 slaughtered cattle, 1,083 (2.27%) have macroscopic *C. bovis* cysts. The infection rates in local and imported cattle were 1.94% (203/10438) and 2.36% (880/37325), respectively. The prevalence of *C. bovis* infection was found to be significantly associated with the age, sex, and body condition of slaughtered cattle (*p* < 0.05). In the local breed, the heart had the highest occurrence of *C. bovis* (64%), whereas in the imported breed, the masseter muscles were the most susceptible to infection (92.00%). The histopathological analysis demonstrated that the deteriorated cysts were situated in regions of tissue coagulative necrosis, characterized by a wide range of inflammatory infiltrates and collagen fibers, particularly eosinophils and macrophages. In addition, the muscle fibers undergo degenerative changes, which may lead to the loss of normal muscle structure. The presence of the *C. bovisHDP2* gene was demonstrated by the PCR analysis of *C. bovis* cyst samples, which showed positive diagnostic bands at 599 bp on gel electrophoresis.

**Conclusions:**

Based on the findings in this study, the slaughtered cattle meant for human consumption in Aswan, Egypt, had *C. bovis* lesions restricted to one or a few organs. The study revealed that the spread of infection in these valuable organs in cattle has negative implications for food safety.

**Recommendation:**

We strongly support the One Health strategy for preventing zoonotic pathogens from spreading to humans and preventing economic loss in cattle production.

**Supplementary Information:**

The online version contains supplementary material available at 10.1186/s12917-025-04768-y.

## Introduction

Bovine cysticercosis is among the communicable infections regarded to be of socioeconomic and public health relevance in numerous nations and has an impact on international commerce in animals and animal products, according to the World Health Organization [1]. The disease is caused by *Cysticercus bovis* (*C. bovis*), which is the larval stage of *Taenia saginata* that inhabits the small intestine of human. In the two-host life cycle, humans are the definitive hosts, and cattle are the intermediate hosts [2]. Human taeniasis is a food born parasite zoonosis that is contracted through eating raw or undercooked meat that contains live cysticercus [3, 4]. Human taeniasis is frequently asymptomatic or marked by minor digestive disorders such as nausea, vomiting, diarrhea, abdominal distress, and weight loss which are occasionally associated with consequences [5]. Both human taeniasis and bovine cysticercosis are widely underappreciated illnesses, most probably because of the lack of clinical indications in cattle, inadequate knowledge of their economic effect, and the fact that human taeniasis has a modest health impact [6].

Crucially, infected individuals can produce millions of eggs every day into the surroundings; these eggs have the potential to live for up to seven months and could be passed to the intermediate host [2]. Usually, cattle contract their infection from grass, grains, or water polluted with eggs [6]. The ingested eggs then hatch in the small intestine to become oncospheres, which pierce the intestinal wall and travel to the muscles via the circulation, where they may develop into infected cysts [2]. Humans come to be infested by consuming raw or undercooked meat with *C. bovis*. Masseter muscles, tongue, heart, diaphragm, and other skeletal muscles are the most frequently preferred sites for *C. bovis*; it is hardly seen in fat or visceral organs [7]. After a duration that can vary from weeks to years, cysticerci degenerate and then calcify [2]. Infection by these parasites is strongly linked to financial losses in the meat sector. Carcasses with significant infestations have to be condemned. However, mild infestation or localized cysticercosis results in the condemnation of the diseased tissues. In addition, the carcass must be frozen for up to three weeks to kill the parasites [8]. Southwest Asia, the Middle East, and the Mediterranean region are classified as high- endemic regions for cysticercosis infection [9].Several studies have been conducted on the prevalence of *C. bovis* in slaughter cattle in various governorates in Egypt, such as Assiut at 10.8% [2], El Menofia at 0.69% [7], Cairo at 6.31% [8], and Aswan at 7.5% [9].

In regards to the diagnosis of the infection, the parasites are usually found by visual inspection of the cysticerci through the postmortem inspection of carcasses. However, conventional techniques have significant limitations and inadequate diagnostic capabilities [10]. The detection of bovine cysticercosis in Egypt is entirely dependent on a visual analysis of the cut muscles of a carcass at designated predilection areas through the postmortem assessment. Lesions with totally clear, cheesy, or calcified cysts are likely to be *C. bovis* [11]. This approach is unreliable, though, because *cysticerci* might be mistaken for lesions brought on by other species, such as Sarcocystis and Actinobacillus, or by other local changes [12]. Genetic evaluation is more accurate, quick, and sensitive than morphological characteristics [12].

In the meat industry, the hypothesis states that parasite-influenced economic losses are significantly associated with infection by these parasites. Carcasses must be completely condemned if they have heavy infestations. However, light infection or localized cysticercosis leads to the condemnation of the infected parts. We hypothesize a low rate of infection in Aswan governorate due to the increase in the acknowledgment of consumers about food safety and law regulations by the Egyptian authority; although some butchers slaughter outside the abattoirs, there are strict measures on the markets as well as the fact that the price of meat has grown annually and the number of slaughtered cattle has decreased.

The study focused on the prevalence of *C. bovis* in slaughtered cattle in Aswan slaughterhouses in Upper Egypt, especially the imported cattle from Africa in the Abu Simbel slaughterhouse. It gave the recent picture for *C. bovis* infection in the studied area. Therefore, from an epidemiological view, the prevalence of *C. bovis* in slaughtered food animals must be achieved annually, covering all of Egypt. The infection, which is transmitted to humans, has major issues, even infecting a low percentage, and must be controlled based on the recent data on such infection. Thus, the goal of current research was to explain the prevalence of *C. bovis* in cattle slaughtered in Aswan Governorate, Upper Egypt, by using a variety of analytical approaches for the descriptive morphological and histopathological identification of specific cysts. Additionally, molecular analysis was implemented to verify the findings, which could potentially serve as an initial stage in the development of a more reliable approach to determining the impact of *C. bovis* on bovine tissue.

## Materials and methods

### Research plan

**Fig. 1:**
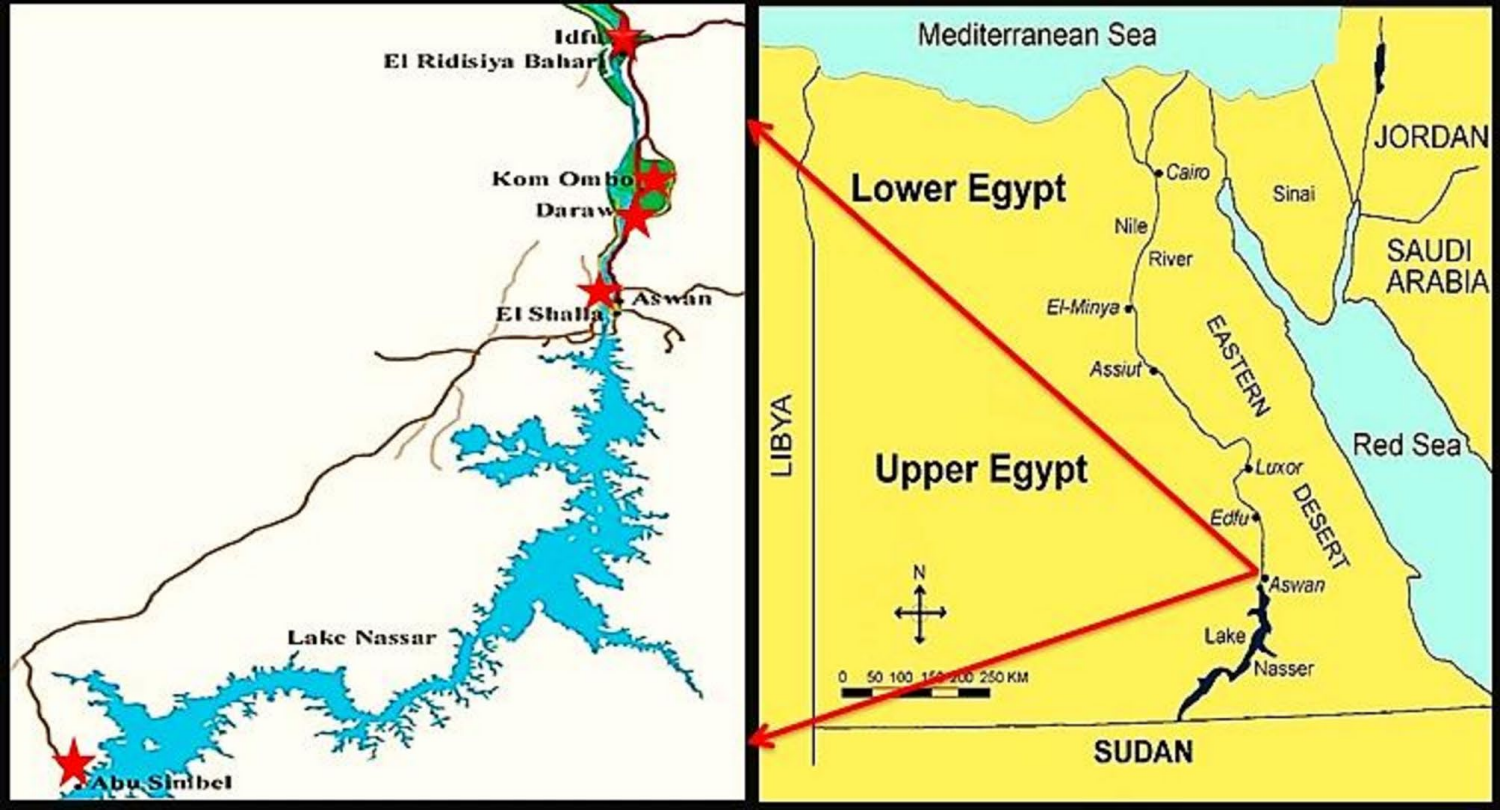
Sampling sites (red star), Aswan map, Egypt

The current cross-sectional study was achieved all over the period from July 2023 to April 2024 at several central slaughterhouse: Edfu (24°58’42.77’’ N and 32°52’32.95’’ E), Kom Ombo (24˚27’8’’ N and 32˚55’42’’ E), Daraw (24°24’24.35” N and 32°55’7.96” E.), Aswan (24°5’20.1768’’ N and 32°53’59.3880’’ E), and Abu Simbel (22°20’12.56’’ N and 31°37’31.91’’ E) in Aswan province, Egypt, to study the prevalence of *C. bovis* in slaughtered cattle intended for human consumption. Aswan, located in southern Egypt, has the hottest summers and covers an area of 62,726 km² (Fig. 1).

Slaughtered cattle were chosen for this study based on an average visit of three days per week that were considered to have a high daily slaughtering rate in the slaughterhouse. The slaughterhouse database archives were used to gather details about animals that had been slaughtered, such as the kind of animal, age, gender, weight, and place of origin (imported or a local breed), as well as the date of the slaughter. The slaughterhouses that were chosen were: (1) the greatest slaughterhouses; (2) selected due to the various geographical origins of cattle; and (3) the high annual cattle throughput. Since they get cattle from different areas, we reasoned that samples from these slaughterhouses would provide a complete consciousness of the *C. bovis* presence in the slaughtered cattle in the research area.

Throughout the postmortem procedure, carcass serial numbers and the positions of cyst organs were noted, and following each sampling day, information about the animals was gathered and recorded from the slaughterhouses database.

### Sample size

The sample size was determined by Thrusfield [13] with a 95% CI and a 5% absolute precision. Accordingly, 34.5% of cattle in Egypt’s Aswan governorate have *C. bovis* cysts [14].

$$\:n=\frac{{Z}^{2}\times\:{P}_{exp}\left(1-{P}_{exp}\right)}{{d}^{2}}$$

n = requisite sample size, Z = appropriate percentage for the standard deviation for the expected confidence = 1.96, P_exp_ = predictable occurrence, and d = anticipated total precision (usually 0.05).

$$\:n=\frac{{1.96\:}^{2}\times\:0.35\left(1-0.35\right)}{{0.05}^{2}}=519\:\left(Minimum\:sample\:size\right)$$

As a result, 47,763 slaughtered cattle were investigated for the occurrence of *C. bovis*, and the greater sample size increased the possibility of finding positive instances.

### Animals

The slaughtered cattle were local or native breeds, whose origin was from Aswan (Baladi and Hagien breeds), and imported breeds that were imported mainly from Sudan and Ethiopia in Africa as meat cattle intended for slaughter according to the Egyptian veterinary legal regulations (Law No. 2128/2011) [15] and slaughtered in the Abu Simbel slaughterhouse following the supervision of Egyptian quarantine veterinarians [16], such as Baggara breeds (Nyalawi, Rizzaki, and Messiri), Kenana breed, and Butana breed. According to the legal requirements of Egyptian abattoirs, routine daily antemortem and postmortem inspections examined about 47,763 slaughtered cattle of various ages and sexes from 10,438 local breeds (8402 male and 2036 female) and 37,325 imported breeds (male) for the prevalence of *C. bovis* lesions. Cattle were categorized as young (between 6 and 24 months, less than two years old), adult (2–5 years old), and elderly (more than five years old) based on their dental eruptions. According to the Egyptian veterinary ministerial Decree No. 72 of 2017, the slaughtering of male cow calves before they reach the age of two years is banned unless their weight reaches 400 kg [17], while a female can be slaughtered only after completing the replacement of all incisors and being above 5 years old (Law No. 517/1986) [18]. Animals were categorized as poor, medium, or good based on their body condition score. Poor animals had hidebound bones and deep-sunk tail bases; medium animals had visible ribs and other bony prominences but a fair, fleshy background when palpated; and good animals had bony structures that were palpable only [19].

### Antemortem and postmortem examination

Every animal submits to a physical clinical checkup before slaughter, adhering to the antemortem assessment protocols [20]. Specifically, superficial lymph nodes, visible mucous membranes, and body states were investigated. The postmortem assessment for *C. bovis* was carried out by the official veterinarian responsible for inspection in the slaughterhouses, following Egyptian rules for bovine examination following Law 517/1986 [18]. The slaughtered cattle were evaluated using standard and detailed visual inspection methods [21]. During this investigation, the routine postmortem inspection consisted of a visual inspection of carcass musculature revealed during dressing and an assessment of the muscular surface exposed by multiple incisions made on the masseter muscles, heart, tongue, diaphragm, and esophagus muscles. If any locations tested positive for *C. bovis*, the infested carcasses were inspected further for shoulders, thighs, and skeletal muscles [3].

The inspected carcass was thoroughly investigated by palpation of the organs before incision; the surface and contents of the tongue were visually evaluated next to a longitudinal ventral incision from the tip of the root. The external and internal masseter muscles were deeply incised parallel to the jaw plane, which is parallel to the jawbone from the lower jaw. The myocardium was visually inspected and cut longitudinally from base to apex. The liver, kidney, lung, biceps, and triceps of the fore and hindquarters, intercostal muscle, and diaphragm were also examined using visualization, palpation, and incision [22]. Probable lesions show whitish-yellow nodules and fluid-filled cysts bulging from the muscle surface (live cysts) or yellow calcified nodules with a gritty sound during trimming (degenerating cysts) [11].

### Microscopic imaging and cyst classification

Every suspected cyst found during the inspection was gathered, labelled, sent to Aswan University, Faculty of Veterinary Medicine, Food Hygiene Laboratory, and preserved at 4 °C till research. Macroscopic analysis and finger palpation helped to classify the suspected *C. bovis* cases as either viable or degenerative. Cysts that were transparent and filled with fluid were deemed viable, whereas those that were empty or included solid or cheesy materials were deemed non-viable or degenerating [11]. The scolex was inspected under a microscope for viability testing via placement in a standard saline solution containing 30% ox bile and incubated for two hours at 32 °C to assess their vitality [23]. In viable cysts, the unarmed scolex typically elapses in 1–2 h [3]. The cysts were subsequently recognized as *C. bovis* by microscopy if they lacked hooks and rostellum on the evaginated scolex with four suckers. Probable cysts were collected and kept in 70% alcohol for further identification [14].

### Histopathological study

For histopathological study, all tissue fragments containing cysts found through the examination were placed in 10% buffered formalin, dried in a series of graded alcohols, cleaned in xylene, fixed in paraffin, cut into sections that were 5 μm thick, and then placed on slides [24]; then the samples were subsequently stained with hematoxylin & eosin and examined microscopically. All samples were thoroughly inspected by highly qualified pathologists at the Faculty of Veterinary Medicine, New Valley University. The results were captured and documented using a Canon digital camera (Canon Powershot A95) attached to a Leitz Dialux 20 Microscope (Germany).

### Molecular analyses

Fifteen probable cysts (11 viable and 4 degenerating) obtained from some suspicious tissue were collected for molecular confirmation: 5 cysts from each of the most predilection sites to the cyst (heart, masseter muscle, and tongue) and represented as 7 samples from Abu Simbel and 2 samples from each of Aswan, Edfu, Kom Ombo, and Daraw. Extraction of *C. bovis* DNA was achieved by the Quick-gDNA™ MiniPrep kit (Cat. No. D3024, Zymoresearch, USA) following the company’s information. Conventional polymerase chain reaction (PCR) was done to identify the *C. bovis HDP2* gene by a forward primer (PTs7S35F1) 5′-CAGTGGCATAGCAGAGGAGGAA-3′ and a reverse primer (PTs7S35R1) 5′-GGACGAAGAATGGAGTTGAAGGT-3′, at 599 bp [25]. The concentration of DNA (25 to 100 ng/µL) was measured by a NanoDrop spectrophotometer (Thermo Scientific, ND8000). PCR amplification was achieved in 25 µL involving 12.5 µL *COSMO* PCR REDMaster Mix (W1020300X, Willowfort Co., UK.), 7.5 µL of nuclease-free water, 1 µL of each primer (10 pmol), and 3 µL of DNA template. The reactions submit to denaturation at 95 °C for 3 min, then 35 cycles of denaturation at 95 °C for 15 s, annealing at 55 °C for 15 s, and extension at 72 °C for 20 s, and a final extension at 72 °C for 10 min. The products were separated on a 1.5% agarose gel and stained with ethidium bromide (0.5 µg/mL), with a 100 bp Plus DNA Ladder^®^ and imaged under UV light (GEL DOC XR). Distilled water was used as a negative control.

### Statistical analysis

The information gathered from the survey of the abattoir was classified and documented. The information was subsequently imported into the Statistical Package for Social Science (SPSS) version 20 for evaluation. Descriptive statistics were utilized to determine the frequency of *C. bovis*, and the Pearson Chi-square Test was employed to assess the association between various factors and *C. bovis* prevalence. The significance threshold and the confidence interval (CI) were set at 5% and 95%, respectively, for all calculations.

## Results

### Prevalence of *C. bovis* in inspected carcasses and related major risk factors

Table 1 displays the characteristics of animals with suspicious *C. bovis* found during postmortem investigations; out of 47,763 examined slaughtered cattle at various central slaughterhouses in Aswan province, Egypt, 2.27% were revealed with macroscopic *C. bovis* cysts. The infection rates were 1.94% (203/10438) and 2.36% (880/37325) in local and imported cattle, respectively. According to the current findings, the male cattle (2.30%) showed a higher prevalence of macroscopic cysts than females (1.38%). The findings showed that the adult and older cattle carcasses had a greater prevalence of macroscopic *C. bovis* infection (2.60% and 1.50%) than younger ones (0.88%). The age, sex, and poor body-conditioned slaughtered cattle were noticed to be considerably linked to the occurrence of *C. bovis* infection (*p* ˂ 0.05). The Aswan (2.31%) and Abu Simbel (2.36%) slaughterhouses found the majority of *C. bovis*-positive cattle carcasses. However, no statistically significant variance was detected in the cattle infection rates (*p* < 0.756) among the slaughterhouses.

**Table 1:** Relative risk factors associated with the prevalence of *C. bovis*

Factors	Examined cattle		Positive for C. bovis lesion			
	No.	%	No.	%	Chi^2∗^	*p*-value
Total No.	47,763	100	1083	2.27		
Breed						< 0.0123
Local	10,438	21.9	203	1.94	6.274	
Imported	37,325	78.1	880	2.36*		
Sex						< 0.0057
Male	45,727	95.7	1055	2.30^*^	7.639	
Female	2036	4.3	28	1.38^*^		
Age					73.087	< 0.0000
young	3643	7.63	32	0.88*		
adult	35,264	73.83	918	2.60^*^		
old	8856	18.54	133	1.50^*^		
BCS					656.108	< 0.0000
Good	32,398	67.83	289	0.89*		
Medium	9755	20.42	476	4.88		
Poor	5610	11.75	318	5.67^*^		
Slaughterhouse						
Edfu	2147	4.5	48	2.24	14.272	< 0.0002
Kom Ombo	2934	6.14	42	1.43		
Daraw	1071	2.24	15	1.4		
Aswan	4286	9	98	2.31		
Abu Simbel	37,325	78.15	880	2.36^*^		

Notes: The percentage of each category was calculated from the total examined animals, while the positive sample percentage was calculated from the total examined animals in each category

* = significantly different by Chi-square statistics at (*p* ˂ 0.05)

### Distribution of *C. bovis* in different tissues

According to the current findings, there were no generalized lesions or widespread of the cyst throughout the carcass. However, localized *C. bovis* lesions mostly restricted to one or a few organs were present in the majority of positive carcasses (Table 2). In the local breed, the heart (64%) was the most predilected organ for *C. bovis*, while in the imported breed; the masseter muscles (92.00%) and tongue (90.23%) were found to be most frequently affected. The anatomical spreading of *C. bovis* showed significant variance (*p* ˂ 0.05) for local breed, while there was no statistically substantial variance (*p* > 0.05) for the imported breed.

**Table 2:** Frequency of *C. bovis* in different tissues of slaughtered cattle

Predilection sites	Total		Frequency of C. bovis			
			Local breed		Imported breed	
	No.	%	No.	%	No.	%
Local cyst*	1588	100	377	23.74	12,151	2.36
Tongue	133	8.38	13	9.78	120	90.23
Masseter muscles	874	55.04	74	8.47	800	92.00*
Heart	202	12.72	129	64.00*	73	36.14
Diaphragm	92	5.80	58	63.00	34	37.00
Oesophagus	128	8.06	39	30.50*	89	70.00*
Forequarter muscles	98	6.20	36	37.00*	62	63.30
Hindquarter muscles	58	3.70	28	48.30	30	51.72
liver	3	0.19	0	0	3	100
*p*-value			< 0.0133		< 0.1497	

Note: *The number of affected organs regardless of the number of positive carcasses as one or several organs may affected on the same carcass

### Morphological identification of *C. bovis* cyst

**Fig. 2:**
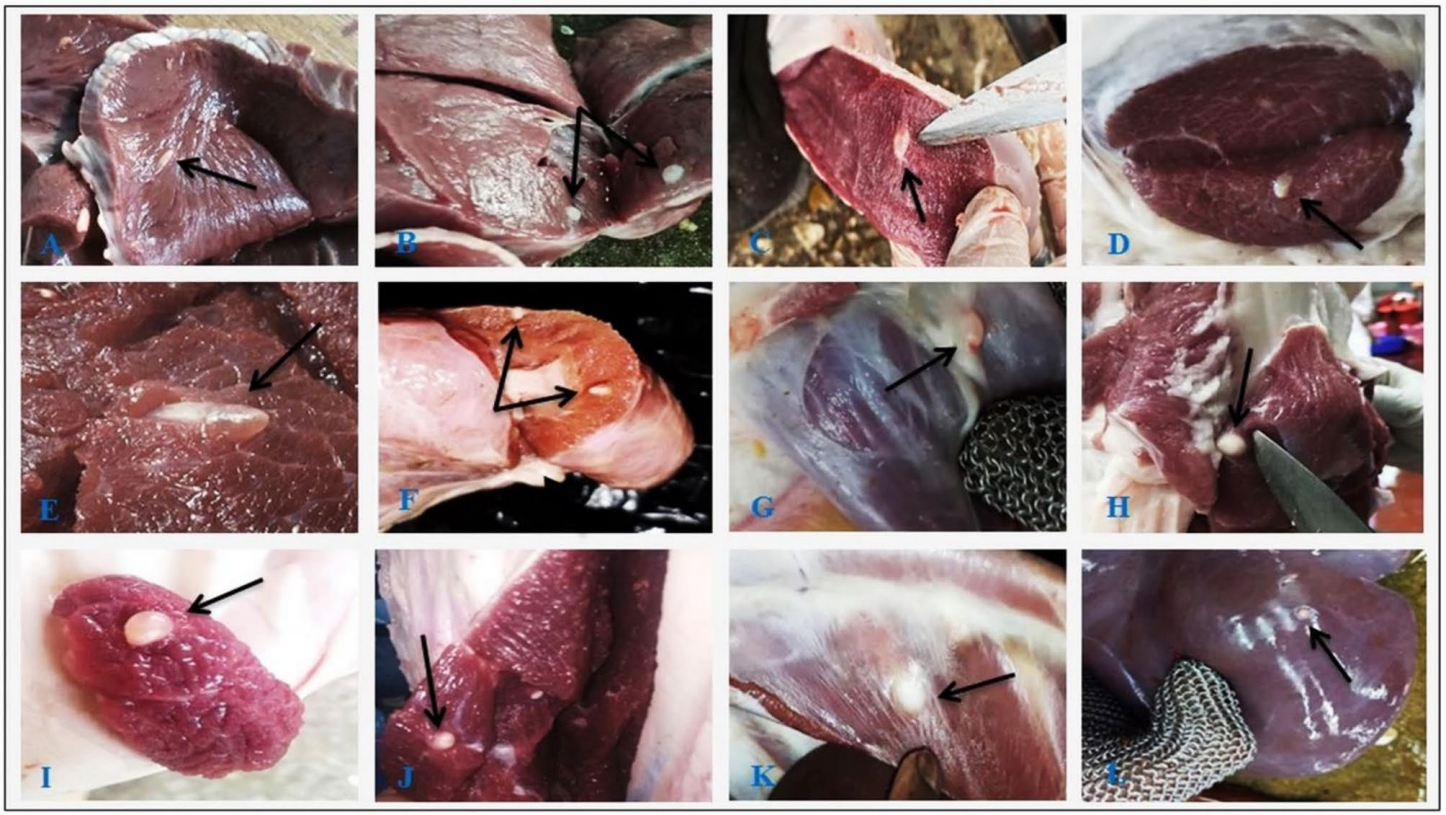
Macroscopic suspected viable *C. bovis* cysts (indicated by black arrowhead) in different tissues of slaughtered cattle showing oval shape, whitish-yellow nodule, fluid-filled cyst protruding from the muscle surface: heart (**A**, **B**), tongue (**C**), masseter muscles (**D**, **E**), esophagus (**F**, **G**), forequarter muscles (**H**, **I**) hindquarter muscles (**J**), diaphragm (**K**), and liver (**L**)

**Fig. 3:**
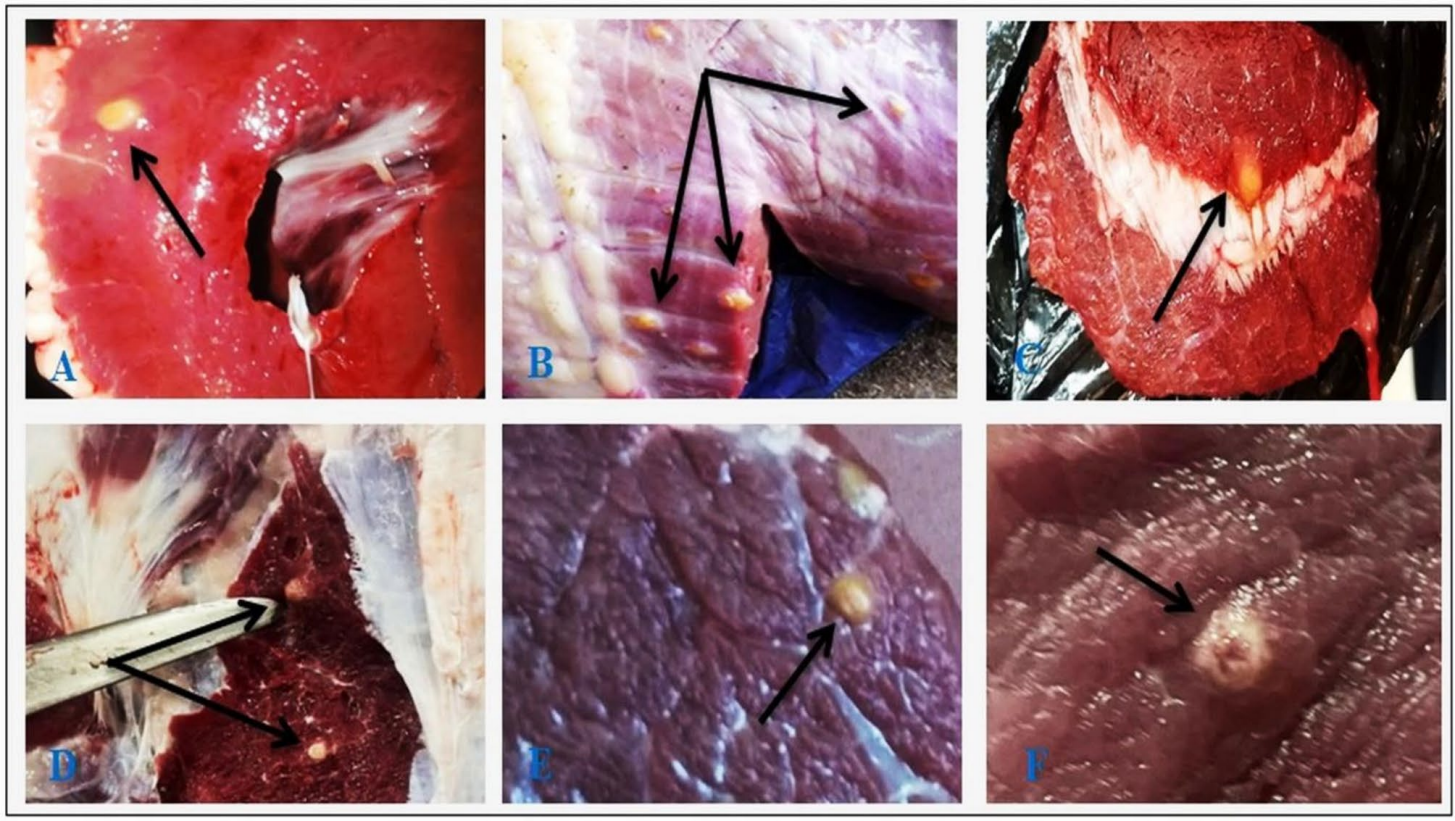
Macroscopic suspected degenerating (non-viable) *C. bovis* cysts (indicated by black arrowhead) in different tissues of slaughtered cattle showing yellow calcified nodule protruding from the muscle surface: heart (**A**, **B**), masseter muscles (**C**, **D**), forequarter muscles (**E**), and hindquarter muscles (**F**)

During gross examination of cattle tissues infected with *C. bovis*, the cysts were classified as either viable or degenerated. Viable cysts were with protoscolex and showed an oval shape, whitish-yellow nodules, and fluid-filled cysts bulging from the muscle surface (Fig. 2). Degenerating (non-viable) cysts were without protoscolex and showed yellow calcified nodules with a gritty sound through cutting (Fig. 3). The severity of the muscle infestation ranged from mild to severe, and the infected animals had cysts in one or a few tissues.

### Histopathological findings

**Fig. 4:**
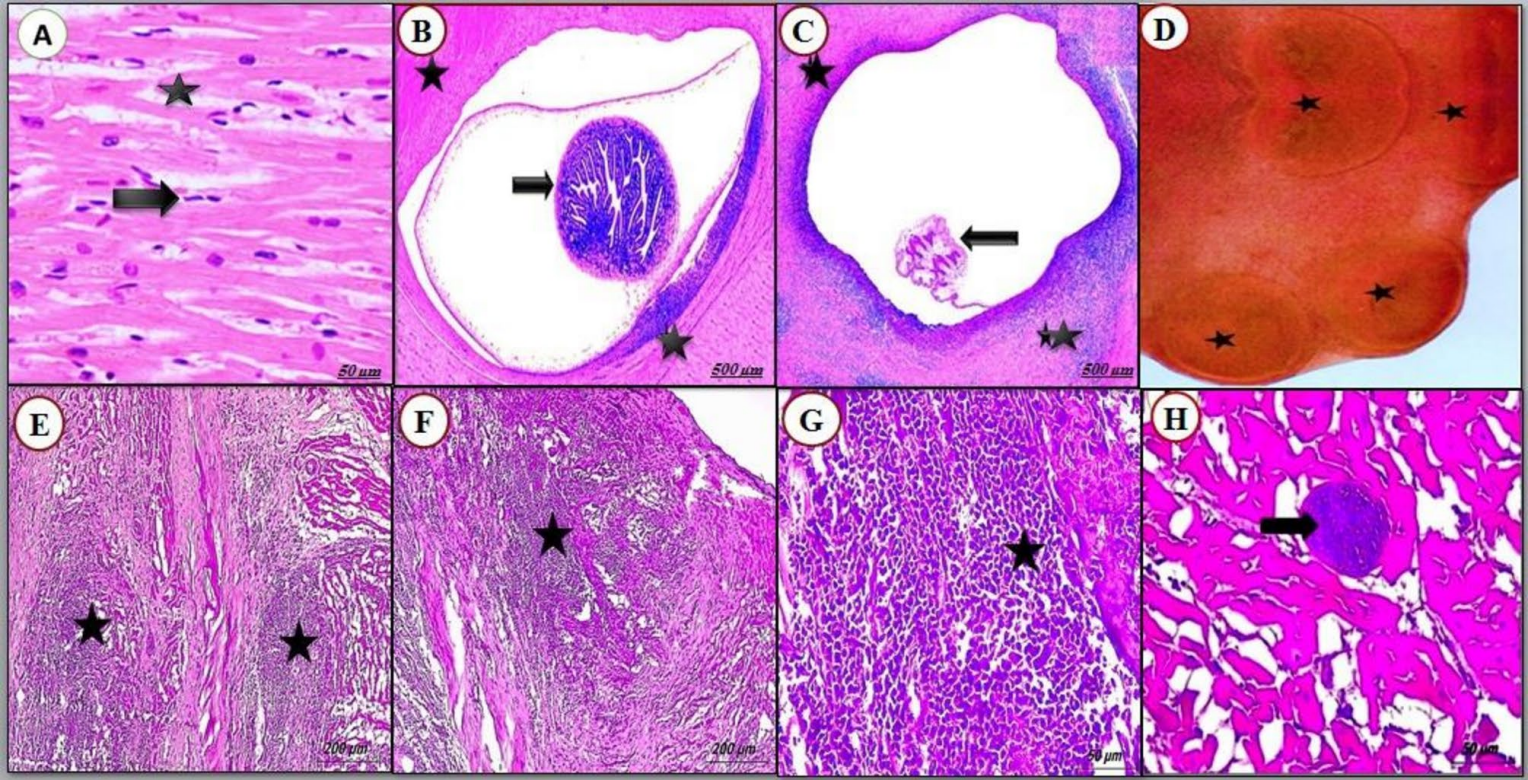
Photomicrograph of cattle heart stained with H & E showing (**A**) normal striated and branching cardiac cells with the nucleus (arrowhead) with perinuclear spaces (star); (**B**) Viable Cysticercus has invaginated scolex with the convoluted spiral canal (arrow) and intense myocardial degeneration (star); (**C**) The cyst formed by a cavity containing the parasite (arrow) surrounded by a fibrous capsule (star) showing inflammatory infiltrate especially eosinophils; (**D**) Microscopic examination *C. bovis* cyst stained by Alum-carmine showing the scolex with four suckers (black star); (**E & F**): severe myocarditis (star) with intense myocardial degeneration; (**G**) higher magnification of previous photo showing inflammatory infiltrate especially eosinophils and macrophages. (**H**): higher magnification of previous photo showing sacs containing the parasite’s larval form

**Fig. 5:**
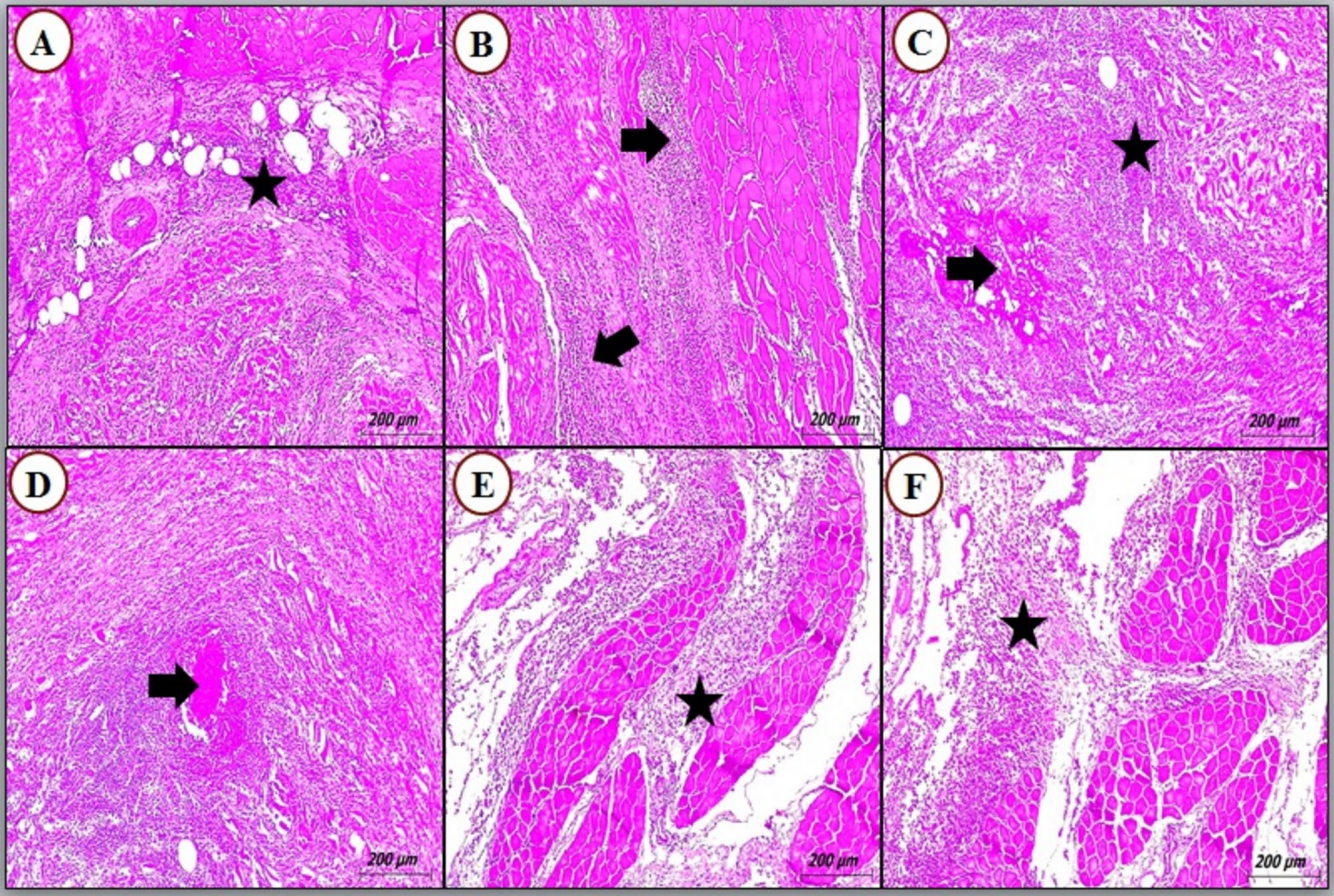
Photomicrograph of cattle skeletal muscles stained with H & E showing (**A**) varying degrees of inflammatory infiltrate and collagen fibers (star); (**B**) intense myositis (arrows); (**C**) severe myositis (arrows) and muscle cells are no longer intact and appear fragmented; (**D**) Granulomatous Inflammation (arrow); (**E & F**) severe inflammatory infiltrations (star) and the muscle fibers undergo degenerative changes, which can result in the loss of normal muscle structure

Microscopically, the cyst was stained red by Alum-carmine stain and the scolex was seen as with four rounded suckers (Fig. 4D). The recovered cyst was a fluid-filled cavity, appearing as fluid-filled sacs containing the parasite’s larval form. The histopathological analysis revealed that degenerated cysts were located in areas of tissue necrosis, characterized by varying degrees of inflammatory infiltration, with a predominance of eosinophils and macrophages, and associated alterations in collagen fiber organization. Also, the muscle fibers undergo degenerative changes, which can result in the loss of normal muscle structure (Figs. 4 and 5).

### **Molecular findings**

**Fig. 6:**
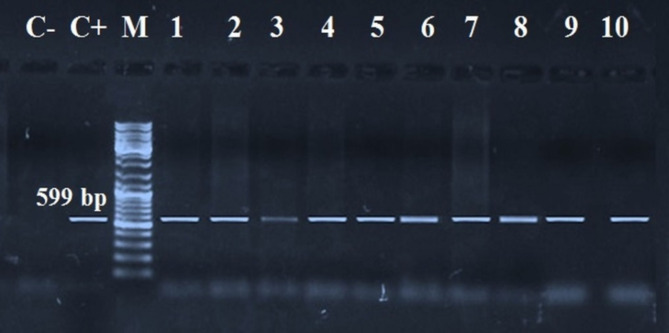
Agarose gel of PCR amplification pattern for *HDP2* gene of *C. bovis* (lane 1 to lane 10) at 599 bp. CN: control negative, CP: control positive, M = Marker (100 bp)

The PCR investigation of *C. bovis* cyst samples had positive diagnostic bands at 599 bp on gel electrophoresis, demonstrating the occurrence of the *C. bovis HDP2* gene in all 15 examined samples (Fig. 6).

## Discussion

Bovine cysticercosis is a significant worldwide distributed disease, particularly in African countries, where it badly affects the economy and public health [11]. Economic losses are usually attributed to the condemnation of massively infested carcasses, condemnation of the infected parts in localized cysticercosis, restriction of exports, and the necessity to store the infected meat in at a temperature not exceeding − 7 °C for period of 3 weeks for keeping the parasites inactivate [25]. The characteristics recognized in our study may be essential for emerging bovine cysticercosis screening and inhibition procedures (Table 1). The present recognition technique relies on the postmortem checkup of carcasses, which has been used as the initial line of investigation of *C. bovis* infection in slaughtered animals according to the Egyptian General Organization of Veterinary Services. A complete postmortem analysis revealed an overall prevalence of 2.27% *C. bovis* among slaughtered cattle in the present investigation. However, the frequency of cysticercosis differs among Aswan’s slaughterhouses; the Abu Simbel slaughterhouse shows a statistically substantially greater prevalence than others in the studied area. Furthermore, it is important to note that the exact causes for the higher prevalence of bovine cysticercosis in Abu Simbel slaughterhouse could be influenced by factors such as high temperature throughout the year. There are many contradictions in seasonal variation between the previous investigations [2]. The variations in infection rate might be due to differences in temperature degree and humidity [11].

The infection prevalence in the current study is higher in imported cattle than in native cattle because imported cattle in Egypt come from severely enzootic African nations with poor hygiene and a lack of infection surveillance organizations. Despite the potential for a higher prevalence of *C. bovis* in the Abu Simbel region due to imported cattle, additional research is necessary. The observed trend could be caused by several factors, such as collecting animals from different places, the weather, the stress that comes from being in overfull and poorly managed areas throughout quarantine, and the chance that infection will be passed from cattle to cattle throughout transport, particularly from watering points. These factors can collectively increase the transmission of diseases within the cattle herd [16].

Both developed and developing nations have observed that animal age is a substantial individual risk factor [26]. The findings support what Kapalamula et al. [27] said; they came up with the idea that there is a link between age and the amount of time someone is exposed to environmental pathogens. This means that older animals are more likely to be at risk because of prolonged contact with the pathogen, which could cause dormant infections to reawaken and a physiological decline in immunity. According to the obtained statistics, the prevalence rate in males is higher than in females, as most of the animals slaughtered were male; also, cattle imported through Abu Simbel quarantine are only males, as the males in Egypt are subject to fattening and meat manufacture, but females are mainly employed for bovine reproduction and milk production. In addition, it is authoritative to note that the slaughter of female cattle below the age of seven is thoroughly forbidden by the regulation of the Egyptian Ministry of Agriculture (laws 53/1966 and 207/1980), excluding some emergency conditions such as trauma and infertility that permit a certificate delivered by the primary veterinary officer [18].

The findings of this research validated the idea that an animal’s bodily state is positively associated with the risk of *C. bovis*. According to Uys et al. [6], this offers more proof that *C. bovis* is a chronic and debilitating disease that causes infected cattle to gradually lose body mass. Several studies have been conducted on the prevalence of *C. bovis* in slaughter cattle in various governorates in Egypt, such as Assiut at 10.8% [2], El Menofia at 0.69% [7], Aswan at 7.5% [9], El-Beheira and El-Gharbia at 4.2% [11], Cairo at 6.31% and 0.86% [8, 12], Beni-Suef and Aswan at 0.34% and 9.1% [14], and El-Minia at 6.6% [28]. The finding of this study indicated that the prevalence of the infection had declined compared to the previous studies by researchers in the studied area [2, 9, 14]. We hypothesize a low rate of infection in Aswan governorate due to the increase in the acknowledgment of consumers about food safety and law regulations by the Egyptian authority; although some butchers slaughter outside the abattoirs, there are strict measures on the markets as well as the fact that the price of meat has grown annually and the number of slaughtered cattle has decreased.

*Cysticercus bovis* is regarded as endemic in numerous regions of Africa; however, the rate of infection in African nations may be higher than recorded due to the absence of appropriate meat assessment and hygiene procedures in several slaughterhouses [7]. The occurrence of *C. bovis* in slaughtered cattle has been reported to be 27.3% in Eastern Ethiopia [23], 3% in Nigeria [29], 26.2% in Tanzania [30], 15% and 22.8% in South Africa [31], 22.6% in Uganda [32], and 2.8% in Ethiopia [33]. The overall *C. bovis* prevalence reported worldwide is as follows: Iran at 0.25% (Khaniki et al., 2009) and Brazil at 5.1% [34]. As well as high numbers of cysticercosis cases were diagnosed in France, Italy, Slovenia, Spain, Denmark, the UK, and Portugal at 0.0002–7.82% [35]. Also, differences in reported prevalence rates could be caused by differences in sample sizes, the way animals are butchered and processed, and the diagnostic methods used in different studies [2].

The anatomical localization of *C. bovis* lesions differs concerning the severity of the infestation; lesions can be limited to one or a few tissues, such as masseter muscles, heart, tongue, diaphragm, and esophagus muscles showing whitish-yellow nodules and fluid-filled cyst bulging from the muscle surface (live cysts) or yellow calcified nodules with a gritty sound during trimming (degenerating cysts). The majority of the influenced cattle in this research had localized *C. bovis* lesions, proposing that the severity of *C. bovis* is lower in cattle slaughtered in Aswan slaughterhouses. The masseter muscle, heart, and tongue in the present study had the highest percentages of impacted predilection sites for *C. bovis*, which confirms the findings of Garedaghi et al. [36] and Mekonnen [37], who found that the cysts favored the masseter, heart, tongue, thigh, and triceps muscles. In contrast, several publications have stated that the parasite has no preference for any specific area [7, 38], although Hailu et al. [39] attribute this to blood kinetics and animal behaviour. The spread of oncospheres is also influenced by any topographical ecological factors influencing the animal’s blood circulation, resulting in fluctuations in the predilection sites throughout meat examination [2].

Moreover, the current finding confirms the results of Jardim et al. [40], who categorized *C. bovis* into four phases. The vesicular phase with a translucent cystic membrane and vesicular fluid with a whitish metacestode in the lowest portion of the vesicle, the colloidal-vesicular phase has an opaque cystic fluid, the nodular granular phase is characterized by cysticercosis lesions encased in white tissue and filled with an amorphous yellowish substance, and the calcified nodular phase is characterized by cysticercosis lesions encased in white tissue and filled with compact non-specific structures. This outcome supports the results noted by Elbayoumi et al. [7], El-Dakhly et al. [14], and Abd El-Hameed et al. [12]. The differences in the frequency of *C. bovis* discovered in preference sites could be explained by the meat inspectors’ proficiency in spotting infested animals, correctly identifying cysts, and using animals for various agricultural tasks that could alter blood kinetics and impact oncosphere distribution. The hot weather in the study area and the place where the cattle were brought in may have affected the eggs’ growth, survival, and the animals’ ability to graze on them. This means that temperature and humidity may have a big impact on the cysticercosis epidemic [11].

According to reports, meat inspection is generally inefficient in diagnosing cysticercosis, especially in nations where the condition is not very common. There been documented false identification of morphologically identical lesions brought on by has different tissue parasites. As a result, the diagnostic accuracy of various approaches, for example, PCR, serology, and histopathology, to identify cysticercosis was assessed [21]. Histopathology of *C. bovis* lesions shows a fluid-filled cavity appearing as fluid-filled sacs containing the parasite’s larval form with the deteriorated cysts located in areas of tissue necrosis showing varying degrees of inflammatory infiltrate and collagen fibers, especially eosinophils and macrophages, and the muscle fibers undergo degenerative changes, which can result in the loss of normal muscle structure.

In the present research, all inflammatory and multinucleated giant cells are identified, signifying cystic lesions and cysticercosis rather than other pathogens. According to the degree of the immune response, the earlier investigations divide the lesions into many stages [41]. Starting with the viable parasite enclosed by a thin layer of collagen fibers, then by the attack of mononucleotide inflammatory cells, these stages culminate in the development of granulomatous tissue with centers containing amorphous materials and the destroyed parasite in a condition of chronic infection [2]. This is consistent with the current findings, which demonstrate that immune cells infiltrate certain lesions that contain a layer of collagen fiber, while other lesions have a high number of fibroblasts and dense layers of connective tissue. Similar outcomes were confirmed by Anwar et al. [2], Elbayoumi et al. [7], and Abdel Aziz et al. [42] Nevertheless, the brief period between the infestation and postmortem procedures may have contributed to the lack of pathology in certain reactor cattle [37].

Conventional PCR was applied as a confirmatory screening in the present work using primers targeted at the *HDP2* gene of isolated DNA from *C. bovis* cysts at 599 bp, resulting in 100% sensitivity and specificity. Similarly, El-Sayed et al. [11], Abd El-Hameed et al. [12], and Gonzalez et al. [43] all employed the same gene. In Egypt, additional studies focused on other genes. Of them, Abdel Aziz et al. [42] created a size of 1600 bp by targeting the *18 S* rDNA gene, while El-Dakhly et al. [14] targeted the *COI* gene at 253 bp. The molecular analysis results verified the cysticercosis prevalence data, which was determined by the examination of infected carcasses. Consequently, the efficacy of cysticercosis recognition in slaughterhouses can be enhanced by integrating PCR with meat evaluation and other assays.

This study demonstrated the endemicity and public health significance of bovine cysticercosis in Aswan, Egypt, particularly within the evaluated slaughterhouses, despite the low prevalence rate. It was shown that the molecular assessment used in this study was more specific for cysticercosis. It can be employed to improve present creativities designed to prevent and control the infection, thereby safeguarding the health of humans and animals. This will make it easier to get reliable data and enhance epidemiological monitoring of the illness. The limitations of this research include the lack of a DNA sequencing technique and phylogenetic studies for molecular validation of *C. bovis*. The study was restricted to slaughtered cattle, did not include other slaughtered animals, and had limited access to the information about the infection in other animals. Furthermore, the study did not involve all slaughterhouses in the studied area; only central slaughterhouses were involved, which may add value to the current results, and these will be taken into consideration in further research.

## Conclusion

Based on post-mortem examination, histopathological, and molecular approaches employed in this study, the slaughtered cattle meant for human consumption in Aswan, Egypt, had *C. bovis* lesions restricted to one or a few organs. This is common in imported cattle at the Abu Simbel slaughterhouse, where it hurts the economy and public health. Further research is needed to determine the actual occurrence of cysticercosis among other herbivores not only in Aswan but in various Egyptian provinces. One health intervention that is coordinated is highly recommended to mitigate the disease’s impact. Also, human consumers must alter their behavior and restrict human sewage from getting to cattle to disrupt *T. saginata*’s life cycle. Using a zoonotic surveillance system along with risk classification for the disease will save money and make it easier to come up with better and more effective ways to control bovine cysticercosis.

## Electronic supplementary material

Below is the link to the electronic supplementary material.

Supplementary Material 1

Supplementary Material 2

Supplementary Material 3

## Data Availability

The manuscript included all our available data. All data were included in the supplementary files.

## Declarations

### Guidelines

All methods were carried out in accordance with relevant guidelines and regulations.
