# Supplementary material for: Cysticercus bovis in slaughtered cattle in upper Egypt: implications for food safety
Source: BMC Vet Res. 2025 May 15;21:344. doi: 10.1186/s12917-025-04768-y (PMC12080167; doi:10.1186/s12917-025-04768-y)
Supplement: Supplementary file 1 — Supplementary Material 1 [file 12917_2025_4768_MOESM1_ESM.docx]

Supplementary file


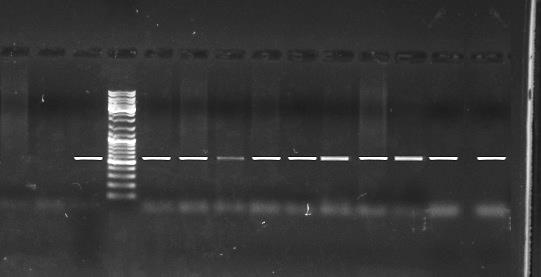


**Figure 5.** Agarose gel of PCR amplification pattern for *HDP2* gene of *C. bovis* (Lane 1 to lane 10) at 599 bp. C-: control negative, C+: control positive, M= Marker (100 bp).
