# Supplementary material for: Cysticercus bovis in slaughtered cattle in upper Egypt: implications for food safety
Source: BMC Vet Res. 2025 May 15;21:344. doi: 10.1186/s12917-025-04768-y (PMC12080167; doi:10.1186/s12917-025-04768-y)
Supplement: Supplementary file 2 — Supplementary Material 2 [file 12917_2025_4768_MOESM2_ESM.docx]

**S-Table 1: Risk factors associated with the prevalence of *C. bovis* in Edfu slaughterhouse**

| Date | No. | | sex | | age | | | BCS | | | Tissue positive for *C. bovis* | | | | | | | |
| --- | --- | --- | --- | --- | --- | --- | --- | --- | --- | --- | --- | --- | --- | --- | --- | --- | --- | --- |
|  | P + | N - | ♂ | ♀ | ˂ 2 | 2-5 | ˃ 5 | G | M | P | T | M | H | D | O | FQ | HQ | L |
| 5-7-2023 |  | 17 |  |  |  |  |  |  |  |  |  |  |  |  |  |  |  |  |
| 6-7-2023 | 1 | 17 | + |  |  | + |  |  |  | + |  | + | + |  |  |  |  |  |
| 7-7-2023 |  | 13 |  |  |  |  |  |  |  |  |  |  |  |  |  |  |  |  |
| 12-7-2023 |  | 9 |  |  |  |  |  |  |  |  |  |  |  |  |  |  |  |  |
| 13-7-2023 | 2 | 15 | ++ |  |  | ++ |  |  | + | + | + | + | + |  |  |  |  |  |
| 14-7-2023 |  | 14 |  |  |  |  |  |  |  |  |  |  |  |  |  |  |  |  |
| 19-7-2023 | 1 | 16 | + |  |  | + |  |  |  | + |  |  |  |  |  |  |  |  |
| 20-7-2023 | 1 | 18 | + |  |  | + |  |  |  | + |  | + |  |  | + |  | + |  |
| 21-7-2023 |  | 15 |  |  |  |  |  |  |  |  |  |  |  |  |  |  |  |  |
| 26-7-2023 |  | 14 |  |  |  |  |  |  |  |  |  |  |  |  |  |  |  |  |
| 27-7-2023 | 1 | 21 |  | + |  |  | + |  | + |  |  |  |  | + | + |  |  |  |
| 28-7-2023 |  | 13 |  |  |  |  |  |  |  |  |  |  |  |  |  |  |  |  |
| 2-8-2023 |  | 18 |  |  |  |  |  |  |  |  |  |  |  |  |  |  |  |  |
| 9-8-2023 | 2 | 16 | ++ |  |  | ++ |  | + |  | + |  |  | + | + |  |  |  |  |
| 10-8-2023 |  | 12 |  |  |  |  |  |  |  |  |  |  |  |  |  |  |  |  |
| 11-8-2023 | 1 | 15 | + |  |  | + |  |  |  | + |  | + | + |  |  | + |  |  |
| 16-8-2023 |  | 17 |  |  |  |  |  |  |  |  |  |  |  |  |  |  |  |  |
| 17-8-2023 | 1 | 18 | + |  | + |  |  |  | + |  |  |  | + |  | + |  |  |  |
| 18-8-2023 |  | 12 |  |  |  |  |  |  |  |  |  |  |  |  |  |  |  |  |
| 23-8-2023 |  | 14 |  |  |  |  |  |  |  |  |  |  |  |  |  |  |  |  |
| 24-8-2023 |  | 15 |  |  |  |  |  |  |  |  |  |  |  |  |  |  |  |  |
| 25-8-2023 | 1 | 20 |  | + |  |  | + |  | + |  |  |  |  | + |  |  | + |  |
| 30-8-2023 |  | 14 |  |  |  |  |  |  |  |  |  |  |  |  |  |  |  |  |
| 31-8-2023 |  | 14 |  |  |  |  |  |  |  |  |  |  |  |  |  |  |  |  |
| 1-9-2023 | 2 | 13 | + | + |  | + | + | + | + |  | + |  | + |  |  | + |  |  |
| 6-9-2023 |  | 21 |  |  |  |  |  |  |  |  |  |  |  |  |  |  |  |  |
| 7-9-2023 | 1 | 16 | + |  |  | + |  |  |  | + |  | + |  | + | + |  |  |  |
| 8-9-2023 |  | 15 |  |  |  |  |  |  |  |  |  |  |  |  |  |  |  |  |
| 13-9-2023 |  | 14 |  |  |  |  |  |  |  |  |  |  |  |  |  |  |  |  |
| 14-9-2023 | 1 | 17 | + |  |  | + |  |  |  | + |  | + | + |  |  |  |  |  |
| 15-9-2023 | 1 | 14 | + |  | + |  |  |  | + |  |  |  |  |  |  |  |  |  |
| 20-9-2023 |  | 22 |  |  |  |  |  |  |  |  |  |  |  |  |  |  |  |  |
| 21-9-2023 |  | 16 |  |  |  |  |  |  |  |  |  |  |  |  |  |  |  |  |
| 22-9-2023 | 1 | 14 | + |  |  |  | + |  | + |  |  |  | + |  |  | + |  |  |
| 27-9-2023 |  | 14 |  |  |  |  |  |  |  |  |  |  |  |  |  |  |  |  |
| 28-9-2023 | 1 | 17 | + |  |  | + |  |  | + |  |  |  | + | + |  |  |  |  |
| 29-9-2023 |  | 22 |  |  |  |  |  |  |  |  |  |  |  |  |  |  |  |  |
| 4-10-2023 | 1 | 17 | + |  |  | + |  |  |  | + |  | + |  |  | + | + |  |  |
| 5-10-2023 |  | 23 |  |  |  |  |  |  |  |  |  |  |  |  |  |  |  |  |
| 6-10-2023 |  | 18 |  |  |  |  |  |  |  |  |  |  |  |  |  |  |  |  |
| 11-10-2023 |  | 16 |  |  |  |  |  |  |  |  |  |  |  |  |  |  |  |  |
| 12-10-2023 | 1 | 18 | + |  |  | + |  |  | + |  |  |  | + |  | + |  |  |  |
| 13-10-2023 |  | 16 |  |  |  |  |  |  |  |  |  |  |  |  |  |  |  |  |
| 18-10-2023 |  | 16 |  |  |  |  |  |  |  |  |  |  |  |  |  |  |  |  |
| 19-10-2023 | 1 | 15 | + |  |  | + |  |  |  | + |  |  | + |  |  | + | + |  |
| 20-10-2023 |  | 14 |  |  |  |  |  |  |  |  |  |  |  |  |  |  |  |  |
| 25-10-2023 |  | 11 |  |  |  |  |  |  |  |  |  |  |  |  |  |  |  |  |
| 26-10-2023 |  | 18 |  |  |  |  |  |  |  |  |  |  |  |  |  |  |  |  |
| 27-10-2023 |  | 24 |  |  |  |  |  |  |  |  |  |  |  |  |  |  |  |  |
| 1-11-2023 | 1 | 15 |  | + |  |  | + |  |  | + | + |  |  | + |  |  |  |  |
| 2-11-2023 |  | 14 |  |  |  |  |  |  |  |  |  |  |  |  |  |  |  |  |
| 3-11-2023 |  | 14 |  |  |  |  |  |  |  |  |  |  |  |  |  |  |  |  |
| 8-11-2023 |  | 13 |  |  |  |  |  |  |  |  |  |  |  |  |  |  |  |  |
| 9-11-2023 |  | 16 |  |  |  |  |  |  |  |  |  |  |  |  |  |  |  |  |
| 10-11-2023 |  | 17 |  |  |  |  |  |  |  |  |  |  |  |  |  |  |  |  |
| 15-11-2023 | 1 | 14 | + |  |  | + |  |  | + |  |  | + |  |  |  | + | + |  |
| 16-11-2023 |  | 15 |  |  |  |  |  |  |  |  |  |  |  |  |  |  |  |  |
| 17-11-2023 |  | 18 |  |  |  |  |  |  |  |  |  |  |  |  |  |  |  |  |
| 22-11-2023 |  | 17 |  |  |  |  |  |  |  |  |  |  |  |  |  |  |  |  |
| 23-11-2023 | 1 | 22 | + |  |  |  | + | + |  |  |  |  | + |  | + |  |  |  |
| 24-11-2023 |  | 15 |  |  |  |  |  |  |  |  |  |  |  |  |  |  |  |  |
| 29-11-2023 |  | 11 |  |  |  |  |  |  |  |  |  |  |  |  |  |  |  |  |
| 30-11-2023 |  | 14 |  |  |  |  |  |  |  |  |  |  |  |  |  |  |  |  |
| 1-12-2023 | 1 | 16 | + |  |  | + |  |  | + |  |  | + |  | + |  |  |  |  |
| 6-12-2023 |  | 18 |  |  |  |  |  |  |  |  |  |  |  |  |  |  |  |  |
| 7-12-2023 |  | 15 |  |  |  |  |  |  |  |  |  |  |  |  |  |  |  |  |
| 8-12-2023 | 2 | 24 | ++ |  |  | ++ |  | + | + |  |  |  | + |  |  | + |  |  |
| 13-12-2023 |  | 20 |  |  |  |  |  |  |  |  |  |  |  |  |  |  |  |  |
| 14-12-2023 | 2 | 18 | + | + |  | + | + |  | + | + |  | + |  |  | + |  |  |  |
| 15-12-2023 | 1 | 17 | + |  |  |  | + |  | + |  |  |  | + |  |  |  | + |  |
| 20-12-2023 |  | 18 |  |  |  |  |  |  |  |  |  |  |  |  |  |  |  |  |
| 21-12-2023 |  | 18 |  |  |  |  |  |  |  |  |  |  |  |  |  |  |  |  |
| 22-12-2023 | 1 | 22 |  |  |  |  |  |  |  | + |  |  | + | + |  |  |  |  |
| 27-12-2023 |  | 20 |  |  |  |  |  |  |  |  |  |  |  |  |  |  |  |  |
| 28-12-2023 |  | 25 |  |  |  |  |  |  |  |  |  |  |  |  |  |  |  |  |
| 29-12-2023 |  | 19 |  |  |  |  |  |  |  |  |  |  |  |  |  |  |  |  |
| 3-1-2024 |  | 16 |  |  |  |  |  |  |  |  |  |  |  |  |  |  |  |  |
| 4-1-2024 | 2 | 14 | ++ |  |  | ++ |  | + | + |  | + | + |  |  |  |  |  |  |
| 5-1-2024 |  | 17 |  |  |  |  |  |  |  |  |  |  |  |  |  |  |  |  |
| 10-1-2024 |  | 13 |  |  |  |  |  |  |  |  |  |  |  |  |  |  |  |  |
| 11-1-2024 | 1 | 14 |  | + |  | + |  |  | + |  |  |  | + | + |  |  |  |  |
| 12-1-2024 |  | 16 |  |  |  |  |  |  |  |  |  |  |  |  |  |  |  |  |
| 17-1-2024 |  | 14 |  |  |  |  |  |  |  |  |  |  |  |  |  |  |  |  |
| 18-1-2024 | 1 | 13 | + |  |  | + |  |  |  | + |  |  | + |  |  |  | + |  |
| 19-1-2024 |  | 16 |  |  |  |  |  |  |  |  |  |  |  |  |  |  |  |  |
| 24-1-2024 | 1 | 18 | + |  |  | + |  |  |  | + |  | + |  | + |  |  |  |  |
| 25-1-2024 |  | 11 |  |  |  |  |  |  |  |  |  |  |  |  |  |  |  |  |
| 26-1-2024 | 1 | 22 | + |  | + |  |  |  |  |  |  |  | + |  |  |  |  |  |
| 31-1-2024 |  | 15 |  |  |  |  |  |  |  |  |  |  |  |  |  |  |  |  |
| 1-2-2024 | 1 | 17 | + |  |  | + |  |  |  | + |  |  | + |  |  |  |  |  |
| 2-2-2024 |  | 16 |  |  |  |  |  |  |  |  |  |  |  |  |  |  |  |  |
| 7-1-2024 | 1 | 13 | + |  |  | + |  |  | + |  |  | + | + |  |  |  |  |  |
| 8-2-2024 |  | 21 |  |  |  |  |  |  |  |  |  |  |  |  |  |  |  |  |
| 9-2-2024 | 1 | 15 | + |  |  | + |  |  |  | + |  |  | + | + |  |  |  |  |
| 14-2-2024 |  | 16 |  |  |  |  |  |  |  |  |  |  |  |  |  |  |  |  |
| 15-2-2024 | 1 | 22 | + |  |  | + |  | + |  |  |  | + |  |  |  |  |  |  |
| 16-2-2024 |  | 20 |  |  |  |  |  |  |  |  |  |  |  |  |  |  |  |  |
| 21-2-2024 | 1 | 15 | + |  |  |  | + |  | + |  |  |  | + |  |  |  |  |  |
| 22-2-2024 |  | 12 |  |  |  |  |  |  |  |  |  |  |  |  |  |  |  |  |
| 23-2-2024 | 1 | 18 | + |  |  | + |  |  |  | + |  | + |  | + |  |  |  |  |
| 28-2-2024 |  | 17 |  |  |  |  |  |  |  |  |  |  |  |  |  |  |  |  |
| 29-2-2024 | 1 | 18 | + |  |  | + |  | + |  |  |  |  | + |  |  |  |  |  |
| 1-3-2024 |  | 15 |  |  |  |  |  |  |  |  |  |  |  |  |  |  |  |  |
| 6-3-2024 |  | 14 |  |  |  |  |  |  |  |  |  |  |  |  |  |  |  |  |
| 7-3-2024 | 1 | 17 | + |  |  | + |  |  | + |  |  | + | + |  |  |  |  |  |
| 8-3-2024 |  | 15 |  |  |  |  |  |  |  |  |  |  |  |  |  |  |  |  |
| 13-3-2024 | 1 | 18 | + |  |  | + |  |  |  | + |  | + |  | + |  |  |  |  |
| 14-3-2024 |  | 15 |  |  |  |  |  |  |  |  |  |  |  |  |  |  |  |  |
| 15-3-2024 |  | 13 |  |  |  |  |  |  |  |  |  |  |  |  |  |  |  |  |
| 20-3-2024 |  | 22 |  |  |  |  |  |  |  |  |  |  |  |  |  |  |  |  |
| 21-3-2024 | 1 | 18 | + |  |  | + |  |  |  | + |  |  | + |  |  |  |  |  |
| 22-3-2024 |  | 13 |  |  |  |  |  |  |  |  |  |  |  |  |  |  |  |  |
| 27-3-2024 |  | 10 |  |  |  |  |  |  |  |  |  |  |  |  |  |  |  |  |
| 28-3-2024 |  | 21 |  |  |  |  |  |  |  |  |  |  |  |  |  |  |  |  |
| 29-3-2024 |  | 14 |  |  |  |  |  |  |  |  |  |  |  |  |  |  |  |  |
| 3-4-2024 |  | 11 |  |  |  |  |  |  |  |  |  |  |  |  |  |  |  |  |
| 4-4-2024 |  | 15 |  |  |  |  |  |  |  |  |  |  |  |  |  |  |  |  |
| 5-4-2024 |  | 16 |  |  |  |  |  |  |  |  |  |  |  |  |  |  |  |  |
| 10-4-2024 |  | 14 |  |  |  |  |  |  |  |  |  |  |  |  |  |  |  |  |
| 11-4-2024 |  | 21 |  |  |  |  |  |  |  |  |  |  |  |  |  |  |  |  |
| 12-4-2024 | 1 | 18 | + |  |  | + |  |  |  | + |  | + |  |  |  |  |  |  |
| 17-4-2024 |  | 18 |  |  |  |  |  |  |  |  |  |  |  |  |  |  |  |  |
| 18-4-2024 |  | 12 |  |  |  |  |  |  |  |  |  |  |  |  |  |  |  |  |
| 19-4-2024 |  | 12 |  |  |  |  |  |  |  |  |  |  |  |  |  |  |  |  |
| 24-4-2024 |  | 19 |  |  |  |  |  |  |  |  |  |  |  |  |  |  |  |  |
| 25-4-2024 |  | 14 |  |  |  |  |  |  |  |  |  |  |  |  |  |  |  |  |
| 26-4-2024 |  | 15 |  |  |  |  |  |  |  |  |  |  |  |  |  |  |  |  |
| 3-4-2024 |  | 16 |  |  |  |  |  |  |  |  |  |  |  |  |  |  |  |  |
| 3-4-2024 |  | 21 |  |  |  |  |  |  |  |  |  |  |  |  |  |  |  |  |
| **Total no.** | 48 | 2099 | 42 | 6 | 3 | 37 | 8 | 7 | 19 | 22 | 4 | 18 | 25 | 13 | 8 | 7 | 6 | 0 |

BCS: body condition score; G: good, M: medium, P: poor

Tissue positive for *C. bovis*; T: tongue, M: masseter muscle, H: heart, D: diaphragm, O: oesophagus, FQ: forequarter, HQ: hind quarter, L: liver

+: represented one animal; ++: represented two animals

**S-Table 2: Risk factors associated with the prevalence of *C. bovis* in Kom Ombo slaughterhouse**

| Date | No. | | sex | | age | | | BCS | | | Tissue positive for *C. bovis* | | | | | | | |
| --- | --- | --- | --- | --- | --- | --- | --- | --- | --- | --- | --- | --- | --- | --- | --- | --- | --- | --- |
|  | P + | N - | ♂ | ♀ | ˂ 2 | 2-5 | ˃ 5 | G | M | P | T | M | H | D | O | FQ | HQ | L |
| 5-7-2023 |  | 14 |  |  |  |  |  |  |  |  |  |  |  |  |  |  |  |  |
| 6-7-2023 |  | 27 |  |  |  |  |  |  |  |  |  |  |  |  |  |  |  |  |
| 7-7-2023 |  | 24 |  |  |  |  |  |  |  |  |  |  |  |  |  |  |  |  |
| 12-7-2023 | 1 | 14 | + |  |  | + |  |  |  | + |  | + |  |  |  |  |  |  |
| 13-7-2023 |  | 22 |  |  |  |  |  |  |  |  |  |  |  |  |  |  |  |  |
| 14-7-2023 |  | 26 |  |  |  |  |  |  |  |  |  |  |  |  |  |  |  |  |
| 19-7-2023 | 1 | 31 | + |  |  | + |  |  |  | + |  |  | + | + |  |  |  |  |
| 20-7-2023 |  | 22 |  |  |  |  |  |  |  |  |  |  |  |  |  |  |  |  |
| 21-7-2023 |  | 22 |  |  |  |  |  |  |  |  |  |  |  |  |  |  |  |  |
| 26-7-2023 | 1 | 27 | + |  |  | + |  |  | + |  |  |  | + |  |  |  |  |  |
| 27-7-2023 | 1 | 24 | + |  |  | + |  |  |  | + |  |  | + |  | + |  | + |  |
| 28-7-2023 |  | 22 |  |  |  |  |  |  |  |  |  |  |  |  |  |  |  |  |
| 2-8-2023 |  | 18 |  |  |  |  |  |  |  |  |  |  |  |  |  |  |  |  |
| 9-8-2023 | 1 | 16 | + |  |  | + |  | + |  |  |  |  | + | + |  | + |  |  |
| 10-8-2023 |  | 34 |  |  |  |  |  |  |  |  |  |  |  |  |  |  |  |  |
| 11-8-2023 |  | 15 |  |  |  |  |  |  |  |  |  |  |  |  |  |  |  |  |
| 16-8-2023 | 1 | 17 |  | + |  |  | + |  |  | + | + |  | + |  | + |  |  |  |
| 17-8-2023 |  | 25 |  |  |  |  |  |  |  |  |  |  |  |  |  |  |  |  |
| 18-8-2023 | 1 | 27 | + |  |  | + |  |  | + |  |  |  | + | + |  | + | + |  |
| 23-8-2023 |  | 26 |  |  |  |  |  |  |  |  |  |  |  |  |  |  |  |  |
| 24-8-2023 |  | 24 |  |  |  |  |  |  |  |  |  |  |  |  |  |  |  |  |
| 25-8-2023 | 1 | 25 | + |  |  |  | + |  |  | + |  | + | + |  | + |  |  |  |
| 30-8-2023 |  | 26 |  |  |  |  |  |  |  |  |  |  |  |  |  |  |  |  |
| 31-8-2023 |  | 22 |  |  |  |  |  |  |  |  |  |  |  |  |  |  |  |  |
| 1-9-2023 | 1 | 24 |  | + |  |  | + |  |  | + |  |  | + | + |  | + |  |  |
| 6-9-2023 |  | 21 |  |  |  |  |  |  |  |  |  |  |  |  |  |  |  |  |
| 7-9-2023 |  | 22 |  |  |  |  |  |  |  |  |  |  |  |  |  |  |  |  |
| 8-9-2023 |  | 26 |  |  |  |  |  |  |  |  |  |  |  |  |  |  |  |  |
| 13-9-2023 | 1 | 22 | + |  |  | + |  |  |  | + |  |  | + |  |  | + | + |  |
| 14-9-2023 |  | 26 |  |  |  |  |  |  |  |  |  |  |  |  |  |  |  |  |
| 15-9-2023 |  | 26 |  |  |  |  |  |  |  |  |  |  |  |  |  |  |  |  |
| 20-9-2023 | 1 | 27 | + |  |  | + |  |  | + |  |  |  | + | + |  |  |  |  |
| 21-9-2023 |  | 24 |  |  |  |  |  |  |  |  |  |  |  |  |  |  |  |  |
| 22-9-2023 |  | 22 |  |  |  |  |  |  |  |  |  |  |  |  |  |  |  |  |
| 27-9-2023 | 1 | 22 | + |  |  | + |  |  |  | + |  |  | + |  | + |  |  |  |
| 28-9-2023 |  | 24 |  |  |  |  |  |  |  |  |  |  |  |  |  |  |  |  |
| 29-9-2023 |  | 21 |  |  |  |  |  |  |  |  |  |  |  |  |  |  |  |  |
| 4-10-2023 | 1 | 26 | + |  |  | + |  |  | + |  |  |  | + |  |  | + | + |  |
| 5-10-2023 |  | 26 |  |  |  |  |  |  |  |  |  |  |  |  |  |  |  |  |
| 6-10-2023 | 1 | 24 |  | + |  |  | + |  |  | + |  |  | + |  | + |  |  |  |
| 11-10-2023 | 2 | 22 | ++ |  | + |  | + | + |  | + |  |  | + | + |  |  | + |  |
| 12-10-2023 |  | 21 |  |  |  |  |  |  |  |  |  |  |  |  |  |  |  |  |
| 13-10-2023 |  | 25 |  |  |  |  |  |  |  |  |  |  |  |  |  |  |  |  |
| 18-10-2023 | 1 | 15 | + |  |  | + |  |  | + |  |  |  | + | + |  | + |  |  |
| 19-10-2023 |  | 16 |  |  |  |  |  |  |  |  |  |  |  |  |  |  |  |  |
| 20-10-2023 |  | 19 |  |  |  |  |  |  |  |  |  |  |  |  |  |  |  |  |
| 25-10-2023 | 2 | 21 | + | + |  | + | + |  |  | ++ |  | + | + |  | + |  | + |  |
| 26-10-2023 |  | 18 |  |  |  |  |  |  |  |  |  |  |  |  |  |  |  |  |
| 27-10-2023 |  | 24 |  |  |  |  |  |  |  |  |  |  |  |  |  |  |  |  |
| 1-11-2023 |  | 22 |  |  |  |  |  |  |  |  |  |  |  |  |  |  |  |  |
| 2-11-2023 | 1 | 25 | + |  | + |  |  |  |  | + |  |  | + |  |  |  |  |  |
| 3-11-2023 |  | 21 |  |  |  |  |  |  |  |  |  |  |  |  |  |  |  |  |
| 8-11-2023 |  | 22 |  |  |  |  |  |  |  |  |  |  |  |  |  |  |  |  |
| 9-11-2023 | 1 | 16 | + |  |  | + |  |  |  | + |  |  | + |  | + |  |  |  |
| 10-11-2023 |  | 22 |  |  |  |  |  |  |  |  |  |  |  |  |  |  |  |  |
| 15-11-2023 |  | 24 |  |  |  |  |  |  |  |  |  |  |  |  |  |  |  |  |
| 16-11-2023 | 1 | 15 |  | + |  |  | + |  | + |  |  |  | + | + |  |  |  |  |
| 17-11-2023 |  | 18 |  |  |  |  |  |  |  |  |  |  |  |  |  |  |  |  |
| 22-11-2023 |  | 17 |  |  |  |  |  |  |  |  |  |  |  |  |  |  |  |  |
| 23-11-2023 | 1 | 22 | + |  |  | + |  |  |  | + |  |  | + |  | + |  | + |  |
| 24-11-2023 |  | 22 |  |  |  |  |  |  |  |  |  |  |  |  |  |  |  |  |
| 29-11-2023 | 1 | 24 | + |  |  | + |  |  |  | + |  | + | + |  |  |  |  |  |
| 30-11-2023 | 1 | 24 | + |  | + |  |  |  |  | + |  |  | + |  | + | + |  |  |
| 1-12-2023 |  | 16 |  |  |  |  |  |  |  |  |  |  |  |  |  |  |  |  |
| 6-12-2023 | 2 | 18 | ++ |  |  | + | + |  | + | + |  | + | + | + |  |  |  |  |
| 7-12-2023 |  | 15 |  |  |  |  |  |  |  |  |  |  |  |  |  |  |  |  |
| 8-12-2023 |  | 24 |  |  |  |  |  |  |  |  |  |  |  |  |  |  |  |  |
| 13-12-2023 |  | 22 |  |  |  |  |  |  |  |  |  |  |  |  |  |  |  |  |
| 14-12-2023 |  | 25 |  |  |  |  |  |  |  |  |  |  |  |  |  |  |  |  |
| 15-12-2023 | 1 | 22 |  | + |  |  | + |  |  | + |  | + |  |  |  |  |  |  |
| 20-12-2023 | 1 | 18 |  | + |  |  | + |  | + |  | + |  | + |  |  |  |  |  |
| 21-12-2023 |  | 18 |  |  |  |  |  |  |  |  |  |  |  |  |  |  |  |  |
| 22-12-2023 |  | 22 |  |  |  |  |  |  |  |  |  |  |  |  |  |  |  |  |
| 27-12-2023 | 1 | 20 | + |  |  |  | + |  |  | + |  |  | + |  | + |  | + |  |
| 28-12-2023 |  | 25 |  |  |  |  |  |  |  |  |  |  |  |  |  |  |  |  |
| 29-12-2023 |  | 23 |  |  |  |  |  |  |  |  |  |  |  |  |  |  |  |  |
| 3-1-2024 |  | 16 |  |  |  |  |  |  |  |  |  |  |  |  |  |  |  |  |
| 4-1-2024 | 1 | 14 | + |  |  | + |  | + |  |  |  |  | + | + |  | + |  |  |
| 5-1-2024 |  | 22 |  |  |  |  |  |  |  |  |  |  |  |  |  |  |  |  |
| 10-1-2024 |  | 27 |  |  |  |  |  |  |  |  |  |  |  |  |  |  |  |  |
| 11-1-2024 |  | 17 |  |  |  |  |  |  |  |  |  |  |  |  |  |  |  |  |
| 12-1-2024 |  | 16 |  |  |  |  |  |  |  |  |  |  |  |  |  |  |  |  |
| 17-1-2024 | 1 | 25 | + |  |  | + |  |  | + |  |  | + | + |  |  |  |  |  |
| 18-1-2024 |  | 22 |  |  |  |  |  |  |  |  |  |  |  |  |  |  |  |  |
| 19-1-2024 |  | 16 |  |  |  |  |  |  |  |  |  |  |  |  |  |  |  |  |
| 24-1-2024 |  | 18 |  |  |  |  |  |  |  |  |  |  |  |  |  |  |  |  |
| 25-1-2024 | 1 | 24 | + |  | + |  |  |  |  | + |  | + |  |  |  |  |  |  |
| 26-1-2024 |  | 22 |  |  |  |  |  |  |  |  |  |  |  |  |  |  |  |  |
| 31-1-2024 | 1 | 21 | + |  |  | + |  |  | + |  |  |  | + |  |  |  |  |  |
| 1-2-2024 |  | 22 |  |  |  |  |  |  |  |  |  |  |  |  |  |  |  |  |
| 2-2-2024 |  | 33 |  |  |  |  |  |  |  |  |  |  |  |  |  |  |  |  |
| 7-1-2024 | 1 | 27 | + |  |  | + |  |  | + |  |  | + | + |  |  | + |  |  |
| 8-2-2024 |  | 21 |  |  |  |  |  |  |  |  |  |  |  |  |  |  |  |  |
| 9-2-2024 |  | 34 |  |  |  |  |  |  |  |  |  |  |  |  |  |  |  |  |
| 14-2-2024 |  | 16 |  |  |  |  |  |  |  |  |  |  |  |  |  |  |  |  |
| 15-2-2024 | 1 | 26 | + |  |  | + |  |  |  | + |  | + |  | + |  |  |  |  |
| 16-2-2024 |  | 21 |  |  |  |  |  |  |  |  |  |  |  |  |  |  |  |  |
| 21-2-2024 |  | 17 |  |  |  |  |  |  |  |  |  |  |  |  |  |  |  |  |
| 22-2-2024 |  | 15 |  |  |  |  |  |  |  |  |  |  |  |  |  |  |  |  |
| 23-2-2024 |  | 31 |  |  |  |  |  |  |  |  |  |  |  |  |  |  |  |  |
| 28-2-2024 | 1 | 26 |  | + |  | + |  |  | + |  |  |  | + | + |  |  |  |  |
| 29-2-2024 |  | 24 |  |  |  |  |  |  |  |  |  |  |  |  |  |  |  |  |
| 1-3-2024 |  | 25 |  |  |  |  |  |  |  |  |  |  |  |  |  |  |  |  |
| 6-3-2024 |  | 21 |  |  |  |  |  |  |  |  |  |  |  |  |  |  |  |  |
| 7-3-2024 |  | 21 |  |  |  |  |  |  |  |  |  |  |  |  |  |  |  |  |
| 8-3-2024 | 1 | 25 | + |  |  |  | + |  | + |  |  | + |  |  |  |  |  |  |
| 13-3-2024 |  | 21 |  |  |  |  |  |  |  |  |  |  |  |  |  |  |  |  |
| 14-3-2024 |  | 22 |  |  |  |  |  |  |  |  |  |  |  |  |  |  |  |  |
| 15-3-2024 |  | 24 |  |  |  |  |  |  |  |  |  |  |  |  |  |  |  |  |
| 20-3-2024 | 1 | 22 | + |  |  | + |  | + |  |  |  |  | + | + |  |  |  |  |
| 21-3-2024 |  | 24 |  |  |  |  |  |  |  |  |  |  |  |  |  |  |  |  |
| 22-3-2024 |  | 27 |  |  |  |  |  |  |  |  |  |  |  |  |  |  |  |  |
| 27-3-2024 | 1 | 20 |  | + |  | + |  |  | + |  |  | + |  | + |  |  |  |  |
| 28-3-2024 |  | 33 |  |  |  |  |  |  |  |  |  |  |  |  |  |  |  |  |
| 29-3-2024 |  | 26 |  |  |  |  |  |  |  |  |  |  |  |  |  |  |  |  |
| 3-4-2024 |  | 22 |  |  |  |  |  |  |  |  |  |  |  |  |  |  |  |  |
| 4-4-2024 |  | 26 |  |  |  |  |  |  |  |  |  |  |  |  |  |  |  |  |
| 5-4-2024 | 1 | 25 | + |  |  | + |  |  | + |  |  |  | + | + |  |  |  |  |
| 10-4-2024 |  | 25 |  |  |  |  |  |  |  |  |  |  |  |  |  |  |  |  |
| 11-4-2024 |  | 22 |  |  |  |  |  |  |  |  |  |  |  |  |  |  |  |  |
| 12-4-2024 |  | 21 |  |  |  |  |  |  |  |  |  |  |  |  |  |  |  |  |
| 17-4-2024 | 1 | 35 | + |  |  | + |  |  |  | + |  | + |  |  |  |  |  |  |
| 18-4-2024 |  | 26 |  |  |  |  |  |  |  |  |  |  |  |  |  |  |  |  |
| 19-4-2024 |  | 16 |  |  |  |  |  |  |  |  |  |  |  |  |  |  |  |  |
| 24-4-2024 |  | 26 |  |  |  |  |  |  |  |  |  |  |  |  |  |  |  |  |
| 25-4-2024 |  | 18 |  |  |  |  |  |  |  |  |  |  |  |  |  |  |  |  |
| 26-4-2024 |  | 25 |  |  |  |  |  |  |  |  |  |  |  |  |  |  |  |  |
| 3-4-2024 |  | 21 |  |  |  |  |  |  |  |  |  |  |  |  |  |  |  |  |
| 3-4-2024 |  | 24 |  |  |  |  |  |  |  |  |  |  |  |  |  |  |  |  |
| **Total no.** | 42 | 2892 | 33 | 9 | 4 | 27 | 11 | 4 | 15 | 23 | 2 | 13 | 32 | 15 | 10 | 9 | 8 | 0 |

BCS: body condition score; G: good, M: medium, P: poor

Tissue positive for *C. bovis*; T: tongue, M: masseter muscle, H: heart, D: diaphragm, O: oesophagus, FQ: forequarter, HQ: hind quarter, L: liver

+: represented one animal; ++: represented two animals

**S-Table 3: Risk factors associated with the prevalence of *C. bovis* in Daraw slaughterhouse**

| Date | No. | | sex | | age | | | BCS | | | Tissue positive for *C. bovis* | | | | | | | |
| --- | --- | --- | --- | --- | --- | --- | --- | --- | --- | --- | --- | --- | --- | --- | --- | --- | --- | --- |
|  | P + | N - | ♂ | ♀ | ˂ 2 | 2-5 | ˃ 5 | G | M | P | T | M | H | D | O | FQ | HQ | L |
| 5-7-2023 |  | 11 |  |  |  |  |  |  |  |  |  |  |  |  |  |  |  |  |
| 6-7-2023 |  | 12 |  |  |  |  |  |  |  |  |  |  |  |  |  |  |  |  |
| 7-7-2023 |  | 8 |  |  |  |  |  |  |  |  |  |  |  |  |  |  |  |  |
| 12-7-2023 |  | 14 |  |  |  |  |  |  |  |  |  |  |  |  |  |  |  |  |
| 13-7-2023 |  | 8 |  |  |  |  |  |  |  |  |  |  |  |  |  |  |  |  |
| 14-7-2023 |  | 9 |  |  |  |  |  |  |  |  |  |  |  |  |  |  |  |  |
| 19-7-2023 |  | 6 |  |  |  |  |  |  |  |  |  |  |  |  |  |  |  |  |
| 20-7-2023 |  | 9 |  |  |  |  |  |  |  |  |  |  |  |  |  |  |  |  |
| 21-7-2023 |  | 5 |  |  |  |  |  |  |  |  |  |  |  |  |  |  |  |  |
| 26-7-2023 |  | 9 |  |  |  |  |  |  |  |  |  |  |  |  |  |  |  |  |
| 27-7-2023 | 1 | 9 | + |  |  | + |  |  |  | + |  | + | + | + |  |  |  |  |
| 28-7-2023 |  | 7 |  |  |  |  |  |  |  |  |  |  |  |  |  |  |  |  |
| 2-8-2023 |  | 5 |  |  |  |  |  |  |  |  |  |  |  |  |  |  |  |  |
| 9-8-2023 |  | 6 |  |  |  |  |  |  |  |  |  |  |  |  |  |  |  |  |
| 10-8-2023 |  | 7 |  |  |  |  |  |  |  |  |  |  |  |  |  |  |  |  |
| 11-8-2023 |  | 9 |  |  |  |  |  |  |  |  |  |  |  |  |  |  |  |  |
| 16-8-2023 |  | 8 |  |  |  |  |  |  |  |  |  |  |  |  |  |  |  |  |
| 17-8-2023 |  | 8 |  |  |  |  |  |  |  |  |  |  |  |  |  |  |  |  |
| 18-8-2023 |  | 11 |  |  |  |  |  |  |  |  |  |  |  |  |  |  |  |  |
| 23-8-2023 |  | 7 |  |  |  |  |  |  |  |  |  |  |  |  |  |  |  |  |
| 24-8-2023 |  | 6 |  |  |  |  |  |  |  |  |  |  |  |  |  |  |  |  |
| 25-8-2023 | 1 | 4 | + |  | + |  |  |  |  | + |  | + | + | + |  |  |  |  |
| 30-8-2023 |  | 3 |  |  |  |  |  |  |  |  |  |  |  |  |  |  |  |  |
| 31-8-2023 |  | 4 |  |  |  |  |  |  |  |  |  |  |  |  |  |  |  |  |
| 1-9-2023 |  | 6 |  |  |  |  |  |  |  |  |  |  |  |  |  |  |  |  |
| 6-9-2023 |  | 7 |  |  |  |  |  |  |  |  |  |  |  |  |  |  |  |  |
| 7-9-2023 | 1 | 8 | + |  |  | + |  |  | + |  |  |  | + | + | + |  |  |  |
| 8-9-2023 |  | 10 |  |  |  |  |  |  |  |  |  |  |  |  |  |  |  |  |
| 13-9-2023 |  | 11 |  |  |  |  |  |  |  |  |  |  |  |  |  |  |  |  |
| 14-9-2023 |  | 7 |  |  |  |  |  |  |  |  |  |  |  |  |  |  |  |  |
| 15-9-2023 |  | 9 |  |  |  |  |  |  |  |  |  |  |  |  |  |  |  |  |
| 20-9-2023 |  | 11 |  |  |  |  |  |  |  |  |  |  |  |  |  |  |  |  |
| 21-9-2023 |  | 8 |  |  |  |  |  |  |  |  |  |  |  |  |  |  |  |  |
| 22-9-2023 | 1 | 5 | + |  |  | + |  |  |  | + |  | + | + | + |  |  | + |  |
| 27-9-2023 |  | 11 |  |  |  |  |  |  |  |  |  |  |  |  |  |  |  |  |
| 28-9-2023 |  | 12 |  |  |  |  |  |  |  |  |  |  |  |  |  |  |  |  |
| 29-9-2023 |  | 8 |  |  |  |  |  |  |  |  |  |  |  |  |  |  |  |  |
| 4-10-2023 | 1 | 10 | + |  |  | + |  |  |  | + |  |  | + | + | + |  |  |  |
| 5-10-2023 |  | 7 |  |  |  |  |  |  |  |  |  |  |  |  |  |  |  |  |
| 6-10-2023 |  | 7 |  |  |  |  |  |  |  |  |  |  |  |  |  |  |  |  |
| 11-10-2023 |  | 9 |  |  |  |  |  |  |  |  |  |  |  |  |  |  |  |  |
| 12-10-2023 |  | 8 |  |  |  |  |  |  |  |  |  |  |  |  |  |  |  |  |
| 13-10-2023 |  | 7 |  |  |  |  |  |  |  |  |  |  |  |  |  |  |  |  |
| 18-10-2023 |  | 6 |  |  |  |  |  |  |  |  |  |  |  |  |  |  |  |  |
| 19-10-2023 |  | 7 |  |  |  |  |  |  |  |  |  |  |  |  |  |  |  |  |
| 20-10-2023 |  | 9 |  |  |  |  |  |  |  |  |  |  |  |  |  |  |  |  |
| 25-10-2023 | 1 | 9 | + |  |  | + |  |  | + |  |  | + |  | + | + |  |  |  |
| 26-10-2023 |  | 9 |  |  |  |  |  |  |  |  |  |  |  |  |  |  |  |  |
| 27-10-2023 |  | 9 |  |  |  |  |  |  |  |  |  |  |  |  |  |  |  |  |
| 1-11-2023 |  | 8 |  |  |  |  |  |  |  |  |  |  |  |  |  |  |  |  |
| 2-11-2023 |  | 7 |  |  |  |  |  |  |  |  |  |  |  |  |  |  |  |  |
| 3-11-2023 |  | 11 |  |  |  |  |  |  |  |  |  |  |  |  |  |  |  |  |
| 8-11-2023 |  | 11 |  |  |  |  |  |  |  |  |  |  |  |  |  |  |  |  |
| 9-11-2023 |  | 7 |  |  |  |  |  |  |  |  |  |  |  |  |  |  |  |  |
| 10-11-2023 | 1 | 10 | + |  |  | + |  |  |  | + | + |  | + | + |  |  |  |  |
| 15-11-2023 |  | 8 |  |  |  |  |  |  |  |  |  |  |  |  |  |  |  |  |
| 16-11-2023 |  | 8 |  |  |  |  |  |  |  |  |  |  |  |  |  |  |  |  |
| 17-11-2023 |  | 7 |  |  |  |  |  |  |  |  |  |  |  |  |  |  |  |  |
| 22-11-2023 |  | 7 |  |  |  |  |  |  |  |  |  |  |  |  |  |  |  |  |
| 23-11-2023 | 1 | 8 | + |  |  | + |  |  |  | + |  | + | + | + |  |  | + |  |
| 24-11-2023 |  | 12 |  |  |  |  |  |  |  |  |  |  |  |  |  |  |  |  |
| 29-11-2023 |  | 5 |  |  |  |  |  |  |  |  |  |  |  |  |  |  |  |  |
| 30-11-2023 |  | 11 |  |  |  |  |  |  |  |  |  |  |  |  |  |  |  |  |
| 1-12-2023 |  | 7 |  |  |  |  |  |  |  |  |  |  |  |  |  |  |  |  |
| 6-12-2023 | 1 | 5 | + |  |  | + |  | + |  |  |  |  | + | + |  | + |  |  |
| 7-12-2023 |  | 9 |  |  |  |  |  |  |  |  |  |  |  |  |  |  |  |  |
| 8-12-2023 |  | 4 |  |  |  |  |  |  |  |  |  |  |  |  |  |  |  |  |
| 13-12-2023 |  | 9 |  |  |  |  |  |  |  |  |  |  |  |  |  |  |  |  |
| 14-12-2023 |  | 6 |  |  |  |  |  |  |  |  |  |  |  |  |  |  |  |  |
| 15-12-2023 |  | 6 |  |  |  |  |  |  |  |  |  |  |  |  |  |  |  |  |
| 20-12-2023 |  | 7 |  |  |  |  |  |  |  |  |  |  |  |  |  |  |  |  |
| 21-12-2023 |  | 8 |  |  |  |  |  |  |  |  |  |  |  |  |  |  |  |  |
| 22-12-2023 | 1 | 4 |  | + |  |  | + |  |  | + |  | + | + | + |  |  |  |  |
| 27-12-2023 |  | 12 |  |  |  |  |  |  |  |  |  |  |  |  |  |  |  |  |
| 28-12-2023 |  | 11 |  |  |  |  |  |  |  |  |  |  |  |  |  |  |  |  |
| 29-12-2023 |  | 9 |  |  |  |  |  |  |  |  |  |  |  |  |  |  |  |  |
| 3-1-2024 |  | 7 |  |  |  |  |  |  |  |  |  |  |  |  |  |  |  |  |
| 4-1-2024 |  | 5 |  |  |  |  |  |  |  |  |  |  |  |  |  |  |  |  |
| 5-1-2024 |  | 9 |  |  |  |  |  |  |  |  |  |  |  |  |  |  |  |  |
| 10-1-2024 |  | 9 |  |  |  |  |  |  |  |  |  |  |  |  |  |  |  |  |
| 11-1-2024 | 1 | 4 | + |  | + |  |  |  |  | + |  | + | + |  | + | + |  |  |
| 12-1-2024 |  | 14 |  |  |  |  |  |  |  |  |  |  |  |  |  |  |  |  |
| 17-1-2024 |  | 9 |  |  |  |  |  |  |  |  |  |  |  |  |  |  |  |  |
| 18-1-2024 |  | 5 |  |  |  |  |  |  |  |  |  |  |  |  |  |  |  |  |
| 19-1-2024 |  | 11 |  |  |  |  |  |  |  |  |  |  |  |  |  |  |  |  |
| 24-1-2024 |  | 7 |  |  |  |  |  |  |  |  |  |  |  |  |  |  |  |  |
| 25-1-2024 | 1 | 7 | + |  |  | + |  |  | + |  |  | + | + | + |  | + |  |  |
| 26-1-2024 |  | 9 |  |  |  |  |  |  |  |  |  |  |  |  |  |  |  |  |
| 31-1-2024 |  | 8 |  |  |  |  |  |  |  |  |  |  |  |  |  |  |  |  |
| 1-2-2024 |  | 5 |  |  |  |  |  |  |  |  |  |  |  |  |  |  |  |  |
| 2-2-2024 |  | 8 |  |  |  |  |  |  |  |  |  |  |  |  |  |  |  |  |
| 7-1-2024 |  | 9 |  |  |  |  |  |  |  |  |  |  |  |  |  |  |  |  |
| 8-2-2024 |  | 6 |  |  |  |  |  |  |  |  |  |  |  |  |  |  |  |  |
| 9-2-2024 |  | 6 |  |  |  |  |  |  |  |  |  |  |  |  |  |  |  |  |
| 14-2-2024 | 1 | 11 | + |  |  |  | + |  |  | + |  | + | + | + | + |  |  |  |
| 15-2-2024 |  | 9 |  |  |  |  |  |  |  |  |  |  |  |  |  |  |  |  |
| 16-2-2024 |  | 7 |  |  |  |  |  |  |  |  |  |  |  |  |  |  |  |  |
| 21-2-2024 |  | 13 |  |  |  |  |  |  |  |  |  |  |  |  |  |  |  |  |
| 22-2-2024 |  | 7 |  |  |  |  |  |  |  |  |  |  |  |  |  |  |  |  |
| 23-2-2024 |  | 9 |  |  |  |  |  |  |  |  |  |  |  |  |  |  |  |  |
| 28-2-2024 |  | 7 |  |  |  |  |  |  |  |  |  |  |  |  |  |  |  |  |
| 29-2-2024 |  | 9 |  |  |  |  |  |  |  |  |  |  |  |  |  |  |  |  |
| 1-3-2024 |  | 10 |  |  |  |  |  |  |  |  |  |  |  |  |  |  |  |  |
| 6-3-2024 |  | 9 |  |  |  |  |  |  |  |  |  |  |  |  |  |  |  |  |
| 7-3-2024 |  | 7 |  |  |  |  |  |  |  |  |  |  |  |  |  |  |  |  |
| 8-3-2024 |  | 9 |  |  |  |  |  |  |  |  |  |  |  |  |  |  |  |  |
| 13-3-2024 | 1 | 4 | + |  |  | + |  |  |  | + |  | + | + | + |  |  |  |  |
| 14-3-2024 |  | 9 |  |  |  |  |  |  |  |  |  |  |  |  |  |  |  |  |
| 15-3-2024 |  | 10 |  |  |  |  |  |  |  |  |  |  |  |  |  |  |  |  |
| 20-3-2024 |  | 6 |  |  |  |  |  |  |  |  |  |  |  |  |  |  |  |  |
| 21-3-2024 |  | 7 |  |  |  |  |  |  |  |  |  |  |  |  |  |  |  |  |
| 22-3-2024 |  | 8 |  |  |  |  |  |  |  |  |  |  |  |  |  |  |  |  |
| 27-3-2024 |  | 10 |  |  |  |  |  |  |  |  |  |  |  |  |  |  |  |  |
| 28-3-2024 |  | 8 |  |  |  |  |  |  |  |  |  |  |  |  |  |  |  |  |
| 29-3-2024 |  | 9 |  |  |  |  |  |  |  |  |  |  |  |  |  |  |  |  |
| 3-4-2024 |  | 11 |  |  |  |  |  |  |  |  |  |  |  |  |  |  |  |  |
| 4-4-2024 |  | 9 |  |  |  |  |  |  |  |  |  |  |  |  |  |  |  |  |
| 5-4-2024 |  | 11 |  |  |  |  |  |  |  |  |  |  |  |  |  |  |  |  |
| 10-4-2024 | 1 | 8 | + |  |  | + |  |  |  | + |  | + | + |  | + |  |  |  |
| 11-4-2024 |  | 9 |  |  |  |  |  |  |  |  |  |  |  |  |  |  |  |  |
| 12-4-2024 |  | 10 |  |  |  |  |  |  |  |  |  |  |  |  |  |  |  |  |
| 17-4-2024 |  | 7 |  |  |  |  |  |  |  |  |  |  |  |  |  |  |  |  |
| 18-4-2024 |  | 8 |  |  |  |  |  |  |  |  |  |  |  |  |  |  |  |  |
| 19-4-2024 |  | 9 |  |  |  |  |  |  |  |  |  |  |  |  |  |  |  |  |
| 24-4-2024 |  | 10 |  |  |  |  |  |  |  |  |  |  |  |  |  |  |  |  |
| 25-4-2024 |  | 8 |  |  |  |  |  |  |  |  |  |  |  |  |  |  |  |  |
| 26-4-2024 |  | 9 |  |  |  |  |  |  |  |  |  |  |  |  |  |  |  |  |
| 3-4-2024 |  | 10 |  |  |  |  |  |  |  |  |  |  |  |  |  |  |  |  |
| 3-4-2024 |  | 9 |  |  |  |  |  |  |  |  |  |  |  |  |  |  |  |  |
| **Total no.** | 15 | 1056 | 14 | 1 | 2 | 11 | 2 | 1 | 3 | 11 | 1 | 11 | 14 | 13 | 6 | 3 | 2 | 0 |

BCS: body condition score; G: good, M: medium, P: poor

Tissue positive for *C. bovis*; T: tongue, M: masseter muscle, H: heart, D: diaphragm, O: oesophagus, FQ: forequarter, HQ: hind quarter, L: liver

+: represented one animal; ++: represented two animals

**S-Table 4: Risk factors associated with the prevalence of *C. bovis* in Aswan slaughterhouse**

| Date | No. | | sex | | age | | | BCS | | | Tissue positive for *C. bovis* | | | | | | | |
| --- | --- | --- | --- | --- | --- | --- | --- | --- | --- | --- | --- | --- | --- | --- | --- | --- | --- | --- |
|  | P + | N - | ♂ | ♀ | ˂ 2 | 2-5 | ˃ 5 | G | M | P | T | M | H | D | O | FQ | HQ | L |
| 5-7-2023 |  | 25 |  |  |  |  |  |  |  |  |  |  |  |  |  |  |  |  |
| 6-7-2023 | 1 | 27 | + |  |  | + |  |  |  | + |  | + | + |  | + |  |  |  |
| 7-7-2023 | 1 | 29 | + |  |  | + |  |  |  | + |  |  | + |  |  | + |  |  |
| 12-7-2023 |  | 27 |  |  |  |  |  |  |  |  |  |  |  |  |  |  |  |  |
| 13-7-2023 | 1 | 29 | + |  |  | + |  |  | + |  |  |  |  |  | + |  |  |  |
| 14-7-2023 |  | 35 | + |  |  | + |  |  |  | + |  | + |  | + |  |  |  |  |
| 19-7-2023 | 1 | 33 | + |  | + | + |  |  |  | + |  |  | + |  |  | + |  |  |
| 20-7-2023 |  | 36 |  |  |  |  |  |  |  |  |  |  |  |  |  |  |  |  |
| 21-7-2023 | 1 | 32 | + |  |  | + |  |  |  | + |  |  | + | + |  | + |  |  |
| 26-7-2023 | 2 | 36 | + | + |  | + | + |  |  | ++ |  | + | + |  | + |  |  |  |
| 27-7-2023 |  | 26 |  |  |  |  |  |  |  |  |  |  |  |  |  |  |  |  |
| 28-7-2023 |  | 22 |  |  |  |  |  |  |  |  |  |  |  |  |  |  |  |  |
| 2-8-2023 |  | 32 |  |  |  |  |  |  |  |  |  |  |  |  |  |  |  |  |
| 9-8-2023 | 2 | 36 | ++ |  |  | ++ |  |  | + | + |  |  | + |  | + |  |  |  |
| 10-8-2023 |  | 37 |  |  |  |  |  |  |  |  |  |  |  |  |  |  |  |  |
| 11-8-2023 | 1 | 33 | + |  |  | + |  |  |  | + |  | + |  | + |  | + |  |  |
| 16-8-2023 | 1 | 32 |  | + |  |  | + |  |  | + |  |  | + |  | + |  |  |  |
| 17-8-2023 |  | 36 |  |  |  |  |  |  |  |  |  |  |  |  |  |  |  |  |
| 18-8-2023 |  | 35 |  |  |  |  |  |  |  |  |  |  |  |  |  |  |  |  |
| 23-8-2023 | 1 | 36 | + |  |  | + |  |  | + |  |  |  | + | + |  |  |  |  |
| 24-8-2023 | 1 | 31 | + |  |  | + |  |  |  | + |  |  |  |  |  |  |  |  |
| 25-8-2023 | 1 | 27 |  | + |  |  | + |  |  | + |  | + | + |  | + | + |  |  |
| 30-8-2023 |  | 32 |  |  |  |  |  |  |  |  |  |  |  |  |  |  |  |  |
| 31-8-2023 |  | 35 |  |  |  |  |  |  |  |  |  |  |  |  |  |  |  |  |
| 1-9-2023 | 1 | 26 | + |  |  | + |  |  |  | + |  |  | + |  |  |  | + |  |
| 6-9-2023 |  | 28 |  |  |  |  |  |  |  |  |  | + | + | + |  | + |  |  |
| 7-9-2023 | 1 | 28 | + |  |  | + |  |  |  | + |  |  |  |  |  |  |  |  |
| 8-9-2023 |  | 22 |  |  |  |  |  |  |  |  |  |  |  |  |  |  |  |  |
| 13-9-2023 | 2 | 26 | ++ |  |  | ++ |  |  |  | ++ |  |  | + |  | + |  |  |  |
| 14-9-2023 | 1 | 43 | + |  |  | + |  |  |  | + |  |  | + |  | + |  |  |  |
| 15-9-2023 | 1 | 32 | + |  | + | + |  |  |  | + |  |  | + |  |  |  |  |  |
| 20-9-2023 |  | 37 |  |  |  |  |  |  |  |  |  |  |  |  |  |  |  |  |
| 21-9-2023 | 1 | 33 |  | + |  |  | + |  | + | + |  | + | + | + |  |  | + |  |
| 22-9-2023 |  | 29 |  |  |  |  |  |  |  |  |  |  |  |  |  |  |  |  |
| 27-9-2023 |  | 30 |  |  |  |  |  |  |  |  |  |  |  |  |  |  |  |  |
| 28-9-2023 |  | 31 |  |  |  |  |  |  |  |  |  |  |  |  |  |  |  |  |
| 29-9-2023 | 1 | 28 | + |  |  | + |  | + |  | + | + | + |  |  | + |  |  |  |
| 4-10-2023 |  | 42 |  |  |  |  |  |  |  |  |  |  |  |  |  |  |  |  |
| 5-10-2023 |  | 35 |  |  |  |  |  |  |  |  |  |  |  |  |  |  |  |  |
| 6-10-2023 | 2 | 33 | ++ |  |  | ++ |  |  |  | ++ |  | + |  | + |  | + |  |  |
| 11-10-2023 |  | 46 |  |  |  |  |  |  |  |  |  |  |  |  |  |  |  |  |
| 12-10-2023 | 1 | 36 | + |  |  | + |  |  | + | + |  | + | + |  | + |  |  |  |
| 13-10-2023 |  | 33 |  |  |  |  |  |  |  |  |  |  |  |  |  |  |  |  |
| 18-10-2023 | 2 | 36 | ++ |  |  | + | + |  |  | + |  | + | + | + |  | + |  |  |
| 19-10-2023 |  | 35 |  |  |  |  |  |  |  |  |  |  |  |  |  |  |  |  |
| 20-10-2023 |  | 35 |  |  |  |  |  |  |  |  |  |  |  |  |  |  |  |  |
| 25-10-2023 | 2 | 34 | ++ |  |  | ++ |  | + | + | + |  | + | + |  | + |  |  |  |
| 26-10-2023 |  | 33 |  |  |  |  |  |  |  |  |  |  |  |  |  |  |  |  |
| 27-10-2023 | 1 | 32 | + |  |  | + |  |  | + |  |  |  | + |  |  |  |  |  |
| 1-11-2023 | 2 | 37 | ++ |  | + | + |  |  | + | + |  | + |  | + |  | + |  |  |
| 2-11-2023 |  | 42 |  |  |  |  |  |  |  |  |  |  |  |  |  |  |  |  |
| 3-11-2023 |  | 33 |  |  |  |  |  |  |  |  |  |  |  |  |  |  |  |  |
| 8-11-2023 | 2 | 27 | ++ |  |  | ++ |  |  |  | ++ |  | + | + | + |  |  | + |  |
| 9-11-2023 | 2 | 25 | + | + |  | + |  |  |  | + |  |  | + |  | + |  |  |  |
| 10-11-2023 |  | 28 |  |  |  |  |  |  |  |  |  |  |  |  |  |  |  |  |
| 15-11-2023 | 1 | 34 | + |  |  | + |  |  | + |  |  |  | + |  | + |  |  |  |
| 16-11-2023 | 2 | 33 | + | + |  |  | + |  |  | + |  |  | ++ | + |  | + |  |  |
| 17-11-2023 | 1 | 36 | + |  |  | + |  | + |  |  |  |  |  |  |  |  |  |  |
| 22-11-2023 | 1 | 27 | + |  |  | + |  |  |  | + |  | + |  |  |  |  |  |  |
| 23-11-2023 |  | 36 |  |  |  |  |  |  |  |  |  |  |  |  |  |  |  |  |
| 24-11-2023 | 1 | 43 | + |  |  | + |  |  |  | + |  | + |  | + | + |  |  |  |
| 29-11-2023 |  | 44 |  |  |  |  |  |  |  |  |  |  |  |  |  |  |  |  |
| 30-11-2023 | 1 | 41 | + |  |  | + |  |  | + |  |  |  | + |  |  |  |  |  |
| 1-12-2023 | 3 | 26 | ++ | + |  | ++ | + |  |  | + | + |  | ++ | + |  |  |  |  |
| 6-12-2023 |  | 33 |  |  |  |  |  |  |  |  |  |  |  |  |  |  |  |  |
| 7-12-2023 | 2 | 36 | ++ |  |  | ++ |  |  | + | + |  |  | + |  |  |  | + |  |
| 8-12-2023 | 1 | 37 | + |  | + |  |  |  |  | + |  | + |  |  |  |  |  |  |
| 13-12-2023 | 1 | 36 | + | + |  |  | + |  |  | + |  |  | + |  | + |  |  |  |
| 14-12-2023 |  | 32 |  |  |  |  |  |  |  |  |  |  |  |  |  |  |  |  |
| 15-12-2023 | 1 | 36 | + |  |  | + |  | + | + |  |  |  | + | + |  | + |  |  |
| 20-12-2023 |  | 42 |  |  |  |  |  |  |  |  |  |  |  |  |  |  |  |  |
| 21-12-2023 | 1 | 21 | + |  |  | + |  |  |  | + |  | + |  |  |  |  |  |  |
| 22-12-2023 | 2 | 32 | ++ | + |  | + | + |  |  | ++ |  |  | ++ |  |  |  | + |  |
| 27-12-2023 |  | 35 |  |  |  |  |  |  |  |  |  |  |  |  |  |  |  |  |
| 28-12-2023 | 1 | 28 | + |  |  |  |  |  |  | + |  |  | + |  |  |  |  |  |
| 29-12-2023 | 3 | 24 | ++ | + |  |  | + |  | + | ++ |  |  | ++ |  |  |  | + |  |
| 3-1-2024 |  | 21 |  |  |  |  |  |  |  |  |  |  |  |  |  |  |  |  |
| 4-1-2024 | 2 | 25 | ++ |  |  | + | + |  | + | + |  | + | ++ | + |  | + |  |  |
| 5-1-2024 |  | 34 |  |  |  |  |  |  |  |  |  |  |  |  |  |  |  |  |
| 10-1-2024 | 1 | 27 | + |  |  | + |  |  | + | + |  |  | + |  |  | + |  |  |
| 11-1-2024 | 2 | 33 | ++ |  |  | ++ |  |  |  | ++ |  | + |  |  |  |  |  |  |
| 12-1-2024 |  | 35 |  |  |  |  |  |  |  |  |  |  |  |  |  |  |  |  |
| 17-1-2024 | 1 | 36 | + |  |  |  | + |  |  | + | + |  | + |  |  | + |  |  |
| 18-1-2024 | 1 | 32 | + |  |  | + |  |  | + |  |  |  | + |  |  |  | + |  |
| 19-1-2024 | 1 | 30 | + |  | + |  |  |  |  | + |  | + |  |  |  |  |  |  |
| 24-1-2024 | 1 | 33 | + |  |  | + |  | + |  |  |  |  | + |  |  |  |  |  |
| 25-1-2024 | 1 | 28 | + |  |  | + |  |  |  | + |  |  | + |  |  |  |  |  |
| 26-1-2024 |  | 21 |  |  |  |  |  |  |  |  |  |  |  |  |  |  |  |  |
| 31-1-2024 | 2 | 32 | ++ |  |  | ++ |  |  |  | ++ |  | + | + |  |  | + |  |  |
| 1-2-2024 | 1 | 32 | + |  |  | + |  | + | + |  |  |  | + |  |  |  |  |  |
| 2-2-2024 |  | 35 |  |  |  |  |  |  |  |  |  |  |  |  |  |  |  |  |
| 7-1-2024 | 2 | 32 | + | + |  |  | + |  |  | + |  | + | ++ | + |  |  | + |  |
| 8-2-2024 |  | 33 |  |  |  |  |  |  |  |  |  |  |  |  |  |  |  |  |
| 9-2-2024 | 2 | 29 | ++ |  |  | + | + |  | + |  |  | + |  |  |  |  |  |  |
| 14-2-2024 |  | 42 |  |  |  |  |  |  |  |  |  |  |  |  |  |  |  |  |
| 15-2-2024 | 1 | 47 | + |  |  | + |  | + |  |  |  |  | + |  |  |  |  |  |
| 16-2-2024 | 1 | 36 | + |  |  | + |  |  | + |  | + | + |  |  |  |  |  |  |
| 21-2-2024 |  | 33 |  |  |  |  |  |  |  |  |  |  |  |  |  |  |  |  |
| 22-2-2024 | 1 | 33 | + |  |  | + |  |  |  |  |  |  |  |  |  |  |  |  |
| 23-2-2024 | 1 | 35 | + |  |  |  | + |  | + | + |  | + |  |  |  |  |  |  |
| 28-2-2024 | 1 | 32 | + |  |  | + |  |  |  | + |  |  | + | + |  |  |  |  |
| 29-2-2024 |  | 36 |  |  |  |  |  |  |  |  |  |  |  |  |  | + |  |  |
| 1-3-2024 | 1 | 31 | + |  |  | + |  |  | + |  |  |  | + |  |  |  |  |  |
| 6-3-2024 | 1 | 36 | + |  |  | + |  |  |  | + |  | + |  |  |  |  |  |  |
| 7-3-2024 |  | 42 |  |  |  |  |  |  |  |  |  |  |  |  |  |  |  |  |
| 8-3-2024 | 2 | 44 | + | + |  | + | + |  |  | ++ |  | + |  |  |  | + |  |  |
| 13-3-2024 |  | 41 |  |  |  |  |  |  |  |  |  |  |  |  |  |  |  |  |
| 14-3-2024 | 2 | 36 | ++ |  |  | ++ |  |  |  | ++ | + |  |  |  |  |  |  |  |
| 15-3-2024 | 1 | 37 | + |  |  | + |  | + | + |  |  |  | + |  |  |  | + |  |
| 20-3-2024 |  | 33 |  |  |  |  |  |  |  |  |  |  |  |  |  |  |  |  |
| 21-3-2024 | 1 | 32 | + |  |  | + |  |  |  | + |  | + | + |  |  |  | + |  |
| 22-3-2024 |  | 27 |  |  |  |  |  |  |  |  |  |  |  |  |  |  |  |  |
| 27-3-2024 | 1 | 27 | + |  |  |  | + |  | + | + |  |  | + |  |  |  |  |  |
| 28-3-2024 |  | 23 |  |  |  |  |  |  |  |  |  |  |  |  |  |  |  |  |
| 29-3-2024 |  | 24 |  |  |  |  |  |  |  |  |  |  |  |  |  |  |  |  |
| 3-4-2024 | 1 | 35 | + |  |  | + |  |  |  | + |  | + | + |  |  |  |  |  |
| 4-4-2024 |  | 26 |  |  |  |  |  |  |  |  |  |  |  |  |  |  |  |  |
| 5-4-2024 |  | 37 |  |  |  |  |  |  |  |  |  |  |  |  |  |  |  |  |
| 10-4-2024 | 2 | 37 | ++ |  |  | ++ |  | + | + |  |  |  | ++ |  |  |  |  |  |
| 11-4-2024 |  | 22 |  |  |  |  |  |  |  |  |  |  |  |  |  |  |  |  |
| 12-4-2024 | 2 | 35 | ++ |  |  | + | + |  |  | ++ | + |  | ++ |  |  |  | + |  |
| 17-4-2024 |  | 35 |  |  |  |  |  |  |  |  |  |  |  |  |  |  |  |  |
| 18-4-2024 | 1 | 33 | + |  | + | + |  |  |  | + |  | + |  |  |  |  |  |  |
| 19-4-2024 | 1 | 33 | + |  |  | + |  | + |  |  |  |  |  |  |  |  |  |  |
| 24-4-2024 | 1 | 26 | + |  |  | + |  |  |  | + |  |  | + |  |  |  |  |  |
| 25-4-2024 |  | 35 |  |  |  |  |  |  |  |  |  |  |  |  |  |  |  |  |
| 26-4-2024 |  | 22 |  |  |  |  |  |  |  |  |  |  |  |  |  |  |  |  |
| 3-4-2024 | 1 | 26 | + |  |  | + |  |  |  | + |  | + |  |  |  |  | + |  |
| 3-4-2024 |  | 25 |  |  |  |  |  |  |  |  |  |  |  |  |  |  |  |  |
| **Total no.** | 98 | 4188 | 86 | 12 | 6 | 73 | 19 | 10 | 24 | 64 | 6 | 32 | 58 | 17 | 15 | 17 | 12 | 0 |

BCS: body condition score; G: good, M: medium, P: poor

Tissue positive for *C. bovis*; T: tongue, M: masseter muscle, H: heart, D: diaphragm, O: oesophagus, FQ: forequarter, HQ: hind quarter, L: liver

+: represented one animal; ++: represented two animals

**S-Table 5: Risk factors associated with the prevalence of *C. bovis* in Abu Simbel slaughterhouse**

| Date | No. | | sex | | age | | | BCS | | | Tissue positive for *C. bovis* | | | | | | | |
| --- | --- | --- | --- | --- | --- | --- | --- | --- | --- | --- | --- | --- | --- | --- | --- | --- | --- | --- |
|  | P + | N - | ♂ | ♀ | ˂ 2 | 2-5 | ˃ 5 | G | M | P | T | M | H | D | O | FQ | HQ | L |
| 5-7-2023 | 8 | 222 | 8 |  |  | 8 |  | 1 | 6 | 1 | 3 | 6 | 3 |  | 1 |  |  |  |
| 6-7-2023 | 6 | 254 | 6 |  |  | 6 |  |  | 5 | 1 | 1 | 5 |  | 1 |  |  |  |  |
| 7-7-2023 | 4 | 276 | 4 |  |  | 4 |  |  | 3 | 1 | 1 | 4 | 1 |  | 2 |  |  |  |
| 12-7-2023 | 8 | 326 | 8 |  | 1 | 7 |  | 1 | 6 | 1 | 3 | 8 |  | 1 |  | 2 |  |  |
| 13-7-2023 | 7 | 301 | 7 |  |  | 7 |  |  | 6 | 1 | 2 | 6 | 2 | 1 |  |  | 1 |  |
| 14-7-2023 | 3 | 374 | 3 |  |  | 3 |  |  | 2 | 1 | 1 | 3 | 1 |  | 2 |  |  |  |
| 19-7-2023 | 9 | 234 | 9 |  |  | 6 | 3 | 2 | 6 | 1 | 4 | 5 |  |  | 2 |  | 1 |  |
| 20-7-2023 | 4 | 286 | 4 |  |  | 4 |  |  | 3 | 1 | 1 | 3 | 1 | 1 |  |  |  |  |
| 21-7-2023 | 3 | 257 | 3 |  |  | 3 |  |  | 2 | 1 | 1 | 3 | 1 | 1 | 1 |  |  |  |
| 26-7-2023 | 8 | 223 | 8 |  |  | 8 |  | 2 | 4 | 2 | 3 | 7 |  | 1 |  |  |  |  |
| 27-7-2023 | 7 | 215 | 7 |  |  | 5 | 2 |  | 7 | 1 |  | 6 | 2 |  | 2 |  | 1 |  |
| 28-7-2023 | 7 | 327 | 7 |  |  | 7 |  |  | 6 | 1 | 2 | 6 |  |  | 2 |  |  |  |
| 2-8-2023 | 4 | 330 | 4 |  |  | 4 |  | 2 | 3 | 1 | 1 | 3 |  | 1 |  | 2 |  |  |
| 9-8-2023 | 6 | 353 | 6 |  |  | 6 |  |  | 5 | 1 |  | 5 |  |  | 2 |  |  |  |
| 10-8-2023 | 1 | 362 | 1 |  |  | 1 |  |  |  | 1 |  | 1 |  | 1 | 1 |  |  |  |
| 11-8-2023 | 5 | 326 | 5 |  |  | 5 |  | 2 | 1 | 2 | 1 | 3 |  |  | 1 |  | 1 |  |
| 16-8-2023 | 8 | 251 | 8 |  |  | 6 | 2 |  | 7 | 1 |  | 7 | 3 | 1 |  | 2 |  |  |
| 17-8-2023 | 6 | 216 | 6 |  | 1 | 5 |  | 1 | 4 | 1 | 1 | 6 |  |  | 2 |  |  |  |
| 18-8-2023 | 5 | 321 | 5 |  |  | 5 |  |  | 4 | 1 | 1 | 5 |  | 1 | 1 |  | 1 |  |
| 23-8-2023 | 4 | 252 | 4 |  |  | 4 |  |  | 2 | 2 |  | 4 |  |  | 1 | 1 |  |  |
| 24-8-2023 | 8 | 287 | 8 |  |  | 8 |  | 1 | 6 | 1 | 1 | 8 | 2 | 1 | 2 | 1 |  |  |
| 25-8-2023 | 9 | 259 | 9 |  |  | 6 | 3 |  | 8 | 1 |  | 9 | 4 |  |  | 1 |  |  |
| 30-8-2023 | 6 | 263 | 6 |  |  | 6 |  | 1 | 4 | 1 | 2 | 6 |  |  | 1 | 1 |  |  |
| 31-8-2023 | 7 | 352 | 7 |  |  | 7 |  | 1 | 4 | 2 | 2 | 7 |  |  |  | 1 |  |  |
| 1-9-2023 | 7 | 384 | 7 |  |  | 7 |  | 1 | 5 | 1 | 1 | 7 |  |  | 1 |  | 1 |  |
| 6-9-2023 | 6 | 333 | 6 |  |  | 6 |  |  | 4 | 2 |  | 6 | 1 |  | 1 |  |  |  |
| 7-9-2023 | 5 | 326 | 5 |  |  | 5 |  | 2 | 3 | 1 | 1 | 5 |  |  | 1 |  |  |  |
| 8-9-2023 | 6 | 351 | 6 |  |  | 5 | 1 |  | 5 | 1 |  | 6 |  |  | 1 |  |  |  |
| 13-9-2023 | 5 | 247 | 5 |  |  | 5 |  | 2 | 1 | 2 |  | 5 | 1 |  | 1 |  | 1 |  |
| 14-9-2023 | 8 | 236 | 8 |  | 1 | 7 |  |  | 6 | 2 | 2 | 7 | 1 |  |  | 1 |  |  |
| 15-9-2023 | 7 | 276 | 7 |  |  | 7 |  | 1 | 5 | 1 | 2 | 6 |  | 1 | 1 |  | 1 |  |
| 20-9-2023 | 5 | 238 | 5 |  |  | 5 |  | 1 | 4 | 1 |  | 5 |  |  | 2 |  |  |  |
| 21-9-2023 | 7 | 248 | 7 |  |  | 7 |  |  | 5 | 2 |  | 7 | 1 |  | 1 |  | 1 |  |
| 22-9-2023 | 8 | 246 | 8 |  |  | 6 | 2 | 1 | 6 | 1 | 3 | 7 |  |  | 1 | 1 |  |  |
| 27-9-2023 | 4 | 263 | 4 |  |  | 4 |  |  | 3 | 1 |  | 4 |  |  | 1 |  |  |  |
| 28-9-2023 | 7 | 327 | 7 |  |  | 7 |  | 2 | 4 | 1 | 1 | 7 |  | 1 |  | 2 | 1 |  |
| 29-9-2023 | 6 | 294 | 6 |  |  | 6 |  |  | 5 | 1 |  | 6 |  |  | 1 |  |  |  |
| 4-10-2023 | 11 | 342 | 11 |  | 1 | 7 | 3 | 3 | 6 | 2 | 3 | 11 | 1 |  | 2 |  | 1 | 1 |
| 5-10-2023 | 11 | 354 | 11 |  |  | 10 | 1 | 3 | 6 | 2 | 2 | 11 | 2 |  | 1 |  |  |  |
| 6-10-2023 | 5 | 374 | 5 |  |  | 5 |  | 1 | 3 | 1 |  | 5 |  |  | 1 |  | 1 |  |
| 11-10-2023 | 7 | 383 | 7 |  |  | 7 |  | 2 | 4 | 1 |  | 7 |  | 1 | 1 |  |  |  |
| 12-10-2023 | 10 | 247 | 10 |  |  | 7 | 3 | 3 | 6 | 1 | 2 | 8 | 1 |  | 1 |  |  |  |
| 13-10-2023 | 12 | 382 | 12 |  | 1 | 8 | 3 | 4 | 3 | 5 | 3 | 12 | 2 |  |  | 1 |  |  |
| 18-10-2023 | 5 | 257 | 5 |  |  | 5 |  | 1 | 1 | 3 |  | 5 |  |  | 1 |  | 1 |  |
| 19-10-2023 | 7 | 222 | 7 |  |  | 7 |  | 1 | 4 | 2 |  | 7 |  | 1 | 1 |  |  |  |
| 20-10-2023 | 8 | 237 | 8 |  |  | 6 | 2 |  | 6 | 2 | 1 | 8 | 2 |  | 1 |  | 1 |  |
| 25-10-2023 | 6 | 241 | 6 |  |  | 6 |  | 1 | 2 | 3 |  | 6 |  | 1 |  | 2 |  |  |
| 26-10-2023 | 8 | 327 | 8 |  |  | 8 |  | 2 | 5 | 1 |  | 8 | 1 |  | 1 |  | 1 |  |
| 27-10-2023 | 9 | 335 | 9 |  |  | 6 | 3 | 1 | 7 | 1 | 1 | 9 | 2 |  | 2 |  |  |  |
| 1-11-2023 | 4 | 329 | 4 |  |  | 4 |  |  | 1 | 3 |  | 4 |  |  | 1 |  |  |  |
| 2-11-2023 | 7 | 376 | 7 |  |  | 7 |  | 1 | 5 | 1 |  | 7 |  | 1 | 1 |  |  |  |
| 3-11-2023 | 10 | 347 | 10 |  | 1 | 8 | 2 | 3 | 6 | 1 | 2 | 10 | 2 |  | 1 |  |  |  |
| 8-11-2023 | 5 | 341 | 5 |  |  | 5 |  | 1 | 1 | 3 |  | 5 |  |  | 1 |  |  |  |
| 9-11-2023 | 6 | 297 | 6 |  |  | 4 | 2 |  | 5 | 1 | 1 | 6 |  | 1 | 1 |  |  |  |
| 10-11-2023 | 7 | 387 | 7 |  |  | 7 |  |  | 5 | 2 |  | 7 |  |  | 1 |  |  |  |
| 15-11-2023 | 6 | 217 | 6 |  |  | 6 |  | 1 | 4 | 1 | 1 | 6 |  | 1 |  | 2 |  |  |
| 16-11-2023 | 8 | 234 | 8 |  |  | 6 | 3 | 2 | 5 | 1 |  | 8 | 1 |  | 1 |  |  |  |
| 17-11-2023 | 3 | 263 | 3 |  |  | 3 |  | 1 |  | 2 |  | 3 | 1 |  |  | 2 |  |  |
| 22-11-2023 | 7 | 233 | 7 |  |  | 7 |  |  | 6 | 1 | 2 | 7 |  |  | 1 |  |  |  |
| 23-11-2023 | 8 | 216 | 8 |  |  | 8 |  | 2 | 5 | 1 |  | 8 |  |  | 1 |  | 1 |  |
| 24-11-2023 | 4 | 227 | 4 |  |  | 4 |  |  | 2 | 2 |  | 4 | 1 |  |  | 1 |  |  |
| 29-11-2023 | 12 | 298 | 12 |  | 1 | 6 | 5 | 3 | 8 | 1 | 3 | 9 | 3 |  | 1 |  |  | 1 |
| 30-11-2023 | 6 | 264 | 6 |  |  | 6 |  | 1 | 4 | 1 |  | 6 |  | 1 | 1 |  | 1 |  |
| 1-12-2023 | 1 | 248 | 1 |  |  | 1 |  |  |  | 1 |  | 1 |  |  | 1 |  |  |  |
| 6-12-2023 | 7 | 238 | 7 |  |  | 7 |  | 1 | 4 | 2 | 1 | 7 |  | 1 | 1 |  |  |  |
| 7-12-2023 | 8 | 264 | 8 |  |  | 6 | 2 | 2 | 5 | 1 | 2 | 8 | 2 |  | 1 |  | 1 |  |
| 8-12-2023 | 7 | 221 | 7 |  |  | 7 |  | 1 | 5 | 2 |  | 5 | 1 |  | 1 |  |  |  |
| 13-12-2023 | 6 | 254 | 6 |  |  | 3 | 3 | 1 | 2 | 3 | 1 | 6 |  | 1 | 1 |  |  |  |
| 14-12-2023 | 5 | 238 | 5 |  |  | 5 |  | 1 | 3 | 1 |  | 5 | 1 |  | 1 |  |  |  |
| 15-12-2023 | 8 | 231 | 8 |  |  | 8 |  | 2 | 5 | 1 | 2 | 7 | 1 |  |  | 2 |  |  |
| 20-12-2023 | 5 | 284 | 5 |  |  | 5 |  | 1 | 2 | 2 |  | 8 |  |  | 1 |  |  |  |
| 21-12-2023 | 9 | 290 | 9 |  |  | 6 | 3 | 2 | 6 | 1 | 3 | 9 | 1 |  | 1 |  | 1 |  |
| 22-12-2023 | 3 | 230 | 3 |  |  | 3 |  |  | 1 | 2 |  | 3 |  | 1 | 1 |  |  |  |
| 27-12-2023 | 6 | 245 | 6 |  |  | 5 | 1 | 2 | 1 | 3 | 1 | 6 |  |  | 1 |  |  |  |
| 28-12-2023 | 14 | 275 | 14 |  | 1 | 13 |  | 9 | 4 | 1 | 4 | 14 | 2 |  |  | 2 |  | 1 |
| 29-12-2023 | 9 | 288 | 9 |  |  | 7 | 2 | 6 | 2 | 1 |  | 9 | 2 | 1 |  |  |  |  |
| 3-1-2024 | 9 | 283 | 9 |  | 1 | 6 | 2 | 5 | 1 | 3 | 2 | 9 |  |  | 1 | 2 | 1 |  |
| 4-1-2024 | 4 | 254 | 4 |  |  | 4 |  | 1 | 1 | 2 |  | 4 |  |  |  | 1 |  |  |
| 5-1-2024 | 7 | 286 | 7 |  |  | 6 | 1 | 5 | 1 | 1 | 1 | 7 | 1 | 1 |  | 2 |  |  |
| 10-1-2024 | 1 | 324 | 1 |  |  | 1 |  |  |  | 1 |  | 1 |  | 1 | 1 |  |  |  |
| 11-1-2024 | 7 | 243 | 7 |  |  | 5 | 2 | 3 | 2 | 2 | 1 | 7 |  | 1 |  |  | 1 |  |
| 12-1-2024 | 7 | 257 | 7 |  |  | 7 |  | 5 | 1 | 1 | 1 | 7 | 1 |  | 1 |  |  |  |
| 17-1-2024 | 4 | 285 | 4 |  |  | 4 |  | 1 | 1 | 2 |  | 4 |  |  |  | 1 | 1 |  |
| 18-1-2024 | 8 | 297 | 8 |  |  | 5 | 3 | 5 | 2 | 1 | 1 | 8 | 2 |  |  | 1 |  |  |
| 19-1-2024 | 6 | 267 | 6 |  |  | 6 |  | 1 | 4 | 1 | 1 | 6 |  |  | 1 |  | 1 |  |
| 24-1-2024 | 9 | 238 | 9 |  | 1 | 8 |  | 6 | 1 | 2 |  | 9 | 1 |  |  | 1 |  |  |
| 25-1-2024 | 8 | 248 | 8 |  |  | 6 | 2 | 2 | 4 | 2 | 2 | 8 | 1 |  |  | 1 |  |  |
| 26-1-2024 | 5 | 261 | 5 |  |  | 5 |  | 1 | 2 | 2 |  | 5 |  |  | 1 |  |  |  |
| 31-1-2024 | 3 | 236 | 3 |  |  | 3 |  |  | 2 | 1 |  | 3 |  |  | 1 |  | 1 |  |
| 1-2-2024 | 7 | 238 | 7 |  |  | 6 | 1 | 5 |  | 2 | 2 | 7 |  | 1 |  | 1 |  |  |
| 2-2-2024 | 3 | 348 | 3 |  |  | 3 |  |  | 2 | 1 |  | 3 |  | 1 |  | 1 | 1 |  |
| 7-1-2024 | 7 | 332 | 7 |  |  | 6 | 1 | 3 | 3 | 1 |  | 7 |  |  |  | 1 |  |  |
| 8-2-2024 | 6 | 396 | 6 |  |  | 6 |  | 2 | 2 | 2 |  | 6 |  |  | 1 |  |  |  |
| 9-2-2024 | 8 | 380 | 8 |  |  | 6 | 2 | 2 | 5 | 1 | 3 | 8 |  |  | 1 |  |  |  |
| 14-2-2024 | 9 | 327 | 9 |  | 1 | 7 | 1 | 6 |  | 3 |  | 9 | 1 |  | 1 | 1 | 1 |  |
| 15-2-2024 | 6 | 260 | 6 |  |  | 6 | 1 | 2 | 2 | 2 |  | 6 | 1 |  |  | 1 |  |  |
| 16-2-2024 | 6 | 255 | 6 |  |  | 6 |  | 3 | 2 | 1 |  | 6 |  |  |  | 1 | 1 |  |
| 21-2-2024 | 4 | 274 | 4 |  |  | 4 |  | 1 | 2 | 1 |  | 4 | 1 |  | 1 |  |  |  |
| 22-2-2024 | 5 | 288 | 5 |  |  | 4 | 1 | 1 | 3 | 1 |  | 5 |  |  |  | 1 |  |  |
| 23-2-2024 | 1 | 354 | 1 |  |  | 1 |  |  |  | 1 |  | 1 |  |  | 1 |  |  |  |
| 28-2-2024 | 10 | 329 | 10 |  | 1 | 7 | 2 | 6 | 2 | 2 | 3 | 10 | 1 |  | 1 |  |  |  |
| 29-2-2024 | 6 | 365 | 6 |  |  | 5 | 1 | 2 | 3 | 1 |  | 6 |  |  |  | 1 | 1 |  |
| 1-3-2024 | 7 | 367 | 7 |  |  | 7 |  | 3 | 2 | 2 |  | 7 |  | 1 |  |  |  |  |
| 6-3-2024 | 13 | 342 | 13 |  | 1 | 12 |  | 8 | 4 | 1 | 3 | 13 | 2 |  | 1 |  |  |  |
| 7-3-2024 | 9 | 253 | 9 |  |  | 7 | 2 | 6 | 2 | 1 | 2 | 4 |  |  |  | 2 | 1 |  |
| 8-3-2024 | 9 | 298 | 9 |  |  | 6 | 3 | 7 |  | 2 |  | 5 |  |  |  | 1 |  |  |
| 13-3-2024 | 6 | 214 | 6 |  |  | 6 |  | 3 |  | 3 |  | 5 |  |  |  | 1 |  |  |
| 14-3-2024 | 9 | 286 | 9 |  |  | 6 | 3 | 4 | 4 | 1 | 2 | 9 |  | 1 | 1 |  | 1 |  |
| 15-3-2024 | 8 | 222 | 8 |  |  | 8 |  | 7 |  | 1 |  | 6 |  |  |  | 2 |  |  |
| 20-3-2024 | 9 | 245 | 9 |  |  | 7 | 2 | 5 | 3 | 1 | 2 | 9 |  |  | 1 |  |  |  |
| 21-3-2024 | 2 | 215 | 2 |  |  | 2 |  |  |  | 2 |  | 2 |  |  |  |  |  |  |
| 22-3-2024 | 7 | 254 | 7 |  |  | 6 | 1 | 3 | 3 | 1 |  | 6 |  |  |  | 1 |  |  |
| 27-3-2024 | 8 | 211 | 8 |  |  | 8 |  | 5 |  | 3 |  | 10 | 1 |  | 1 |  |  |  |
| 28-3-2024 | 13 | 267 | 13 |  | 1 | 7 | 5 | 5 | 6 | 2 | 4 | 3 | 1 |  |  | 2 |  |  |
| 29-3-2024 | 5 | 243 | 5 |  |  | 5 |  | 1 | 2 | 2 |  | 5 |  |  |  |  |  |  |
| 3-4-2024 | 9 | 217 | 9 |  |  | 9 |  | 4 | 4 | 1 | 3 | 4 |  | 1 |  | 2 |  |  |
| 4-4-2024 | 10 | 326 | 10 |  | 1 | 7 | 2 | 5 | 1 | 4 | 1 | 6 |  |  | 1 |  |  |  |
| 5-4-2024 | 7 | 326 | 7 |  |  | 7 |  | 3 | 3 | 1 | 1 | 7 |  |  |  | 1 |  |  |
| 10-4-2024 | 8 | 236 | 8 |  |  | 8 |  | 4 | 2 | 2 |  | 8 | 1 |  |  | 1 |  |  |
| 11-4-2024 | 9 | 344 | 9 |  |  | 9 |  | 5 | 2 | 2 | 2 | 7 |  |  | 1 | 1 |  |  |
| 12-4-2024 | 6 | 214 | 6 |  |  | 6 |  | 2 | 1 | 3 |  | 5 |  | 1 |  |  |  |  |
| 17-4-2024 | 12 | 253 | 12 |  | 1 | 10 | 1 | 5 | 5 | 2 | 3 | 7 | 1 |  |  | 1 |  |  |
| 18-4-2024 | 8 | 246 | 8 |  |  | 8 |  | 5 | 3 | 1 | 2 | 5 |  | 1 |  | 1 |  |  |
| 19-4-2024 | 9 | 275 | 9 |  |  | 9 |  | 4 | 3 | 2 |  | 9 |  |  |  | 1 |  |  |
| 24-4-2024 | 7 | 287 | 7 |  |  | 7 |  | 4 | 1 | 2 |  | 3 |  |  | 1 |  |  |  |
| 25-4-2024 | 12 | 265 | 12 |  | 1 | 8 | 2 | 6 | 5 | 1 | 2 | 6 | 1 |  | 1 |  |  |  |
| 26-4-2024 | 4 | 235 | 4 |  |  | 4 |  | 1 | 2 | 2 |  | 4 |  |  |  | 1 |  |  |
| 3-4-2024 | 5 | 261 | 5 |  |  | 5 |  |  | 4 | 1 |  | 3 |  |  | 1 |  |  |  |
| 3-4-2024 | 9 | 246 | 9 |  |  | 9 |  | 5 | 3 | 1 |  | 7 | 1 |  |  |  |  |  |
| **Total no.** | 880 | 36445 | 880 | 0 | 17 | 770 | 93 | 267 | 415 | 198 | 120 | 800 | 73 | 34 | 89 | 62 | 30 | 3 |

BCS: body condition score; G: good, M: medium, P: poor

Tissue positive for *C. bovis*; T: tongue, M: masseter muscle, H: heart, D: diaphragm, O: oesophagus, FQ: forequarter, HQ: hind quarter, L: liver
