# Supplementary material for: Cysticercus bovis in slaughtered cattle in upper Egypt: implications for food safety
Source: BMC Vet Res. 2025 May 15;21:344. doi: 10.1186/s12917-025-04768-y (PMC12080167; doi:10.1186/s12917-025-04768-y)
Supplement: Supplementary file 3 — Supplementary Material 3 [file 12917_2025_4768_MOESM3_ESM.docx]

**S-Table 1: Summary of the risk factors associated with the prevalence of *C. bovis* in the examined slaughterhouse**

| **slaughterhouse** | **Total NO.** | **No.** | | **sex** | | **age** | | | **BCS** | | | **Tissue positive for *C. bovis*** | | | | | | | |
| --- | --- | --- | --- | --- | --- | --- | --- | --- | --- | --- | --- | --- | --- | --- | --- | --- | --- | --- | --- |
|  |  | **P +** | **N -** | **♂** | **♀** | **˂ 2** | **2-5** | **˃ 5** | **G** | **M** | **P** | **T** | **M** | **H** | **D** | **O** | **FQ** | **HQ** | **L** |
| **Edfu** | 2147 | 48 | 2099 | 1966 | 181 | 154 | 1717 | 276 | 1286 | 628 | 233 | 4 | 18 | 25 | 13 | 8 | 7 | 6 |  |
| **Kom Ombo** | 2934 | 42 | 2892 | 2215 | 719 | 628 | 1531 | 775 | 1747 | 822 | 365 | 2 | 13 | 32 | 15 | 10 | 9 | 8 |  |
| **Daraw** | 1071 | 15 | 1056 | 852 | 219 | 123 | 662 | 286 | 787 | 197 | 87 | 1 | 11 | 14 | 13 | 6 | 3 | 2 |  |
| **Aswan** | 4286 | 98 | 4188 | 3369 | 917 | 892 | 2317 | 1077 | 2771 | 952 | 563 | 6 | 32 | 58 | 17 | 15 | 17 | 12 |  |
| **Abu Simbel** | 37325 | 880 | 36445 | 37325 |  | 1846 | 29037 | 6442 | 25807 | 7156 | 4362 | 120 | 800 | 73 | 34 | 89 | 62 | 30 | 3 |
| **Total no.** | 47763 | 1083 | 46680 | 45727 | 2036 | 3643 | 35264 | 8856 | 32398 | 9755 | 5610 | 133 | 874 | 202 | 92 | 128 | 98 | 58 | 3 |

**S-Table 2: Summary of the risk factors (sex) associated with the prevalence of *C. bovis* in the examined slaughterhouse**

| **slaughterhouse** | **Total positive NO.** | **sex** | |
| --- | --- | --- | --- |
|  |  | **♂** | **♀** |
| **Edfu** | 48 | 42 | 6 |
| **Kom Ombo** | 42 | 33 | 9 |
| **Daraw** | 15 | 14 | 1 |
| **Aswan** | 98 | 86 | 12 |
| **Abu Simbel** | 880 | 880 | - |
| **Total no.** | 1083 | 1055 | 28 |

**S-Table 3: Summary of the risk factors (age) associated with the prevalence of *C. bovis* in the examined slaughterhouse**

| **slaughterhouse** | **Total positive NO.** | **Age** | | |
| --- | --- | --- | --- | --- |
|  |  | **˂ 2** | **2-5** | **˃ 5** |
| **Edfu** | 48 | 3 | 37 | 8 |
| **Kom Ombo** | 42 | 4 | 27 | 11 |
| **Daraw** | 15 | 2 | 11 | 2 |
| **Aswan** | 98 | 6 | 73 | 19 |
| **Abu Simbel** | 880 | 17 | 770 | 93 |
| **Total no.** | 1083 | 32 | 918 | 133 |

**S-Table 4: Summary of the risk factors (BCS) associated with the prevalence of *C. bovis* in the examined slaughterhouse**

| **slaughterhouse** | **Total positive NO.** | **BCS** | | |
| --- | --- | --- | --- | --- |
|  |  | **G** | **M** | **P** |
| **Edfu** | 48 | 7 | 19 | 22 |
| **Kom Ombo** | 42 | 4 | 15 | 23 |
| **Daraw** | 15 | 1 | 3 | 11 |
| **Aswan** | 98 | 10 | 24 | 64 |
| **Abu Simbel** | 880 | 267 | 415 | 198 |
| **Total no.** | 1083 | 289 | 476 | 318 |

BCS: body condition score; G: good, M: medium, P: poor

**S-Table 5: Prevalence of *C. bovis* in the examined Tissues**

| **slaughterhouse** | **Tissue positive for *C. bovis*** | | | | | | | |
| --- | --- | --- | --- | --- | --- | --- | --- | --- |
|  | **T** | **M** | **H** | **D** | **O** | **FQ** | **HQ** | **L** |
| **Edfu** | 4 | 18 | 25 | 13 | 8 | 7 | 6 | 0 |
| **Kom Ombo** | 2 | 13 | 32 | 15 | 10 | 9 | 8 | 0 |
| **Daraw** | 1 | 11 | 14 | 13 | 6 | 3 | 2 | 0 |
| **Aswan** | 6 | 32 | 58 | 17 | 15 | 17 | 12 | 0 |
| **Abu Simbel** | 120 | 800 | 73 | 34 | 89 | 62 | 30 | 3 |
| **Total no.** | 133 | 874 | 202 | 92 | 128 | 98 | 58 | 3 |

Tissue positive for *C. bovis*

T: tongue, M: masseter muscle, H: heart, D: diaphragm, O: oesophagus, FQ: forequarter, HQ: hind quarter, L: liver
